# Entropies of the Classical Dimer Model

**DOI:** 10.3390/e27070693

**Published:** 2025-06-28

**Authors:** John C. Baker, Marilyn F. Bishop, Tom McMullen

**Affiliations:** 1 CACI International Inc., 16480 Commerce Dr., King George, VA 22485-5860, USA; jcbakeriii@gmail.com; 2 Department of Physics, Virginia Commonwealth University, Richmond, VA 23284-2000, USA; 31jtm2@queensu.ca

**Keywords:** entropy, dimer model, trace theorems, pfaffians, cruciform matrices, DNA charge, biological physics

## Abstract

Biological processes often involve the attachment and detachment of extended molecules to substrates. Here, the classical dimer model is used to investigate these geometric effects on the free energy, which governs both the equilibrium state and the reaction dynamics. We present a simplified version of Fisher’s derivation of the partition function of a two-dimensional dimer model at filling factor ν=1, which takes into account the blocking of two adjacent sites by each dimer. Physical consequences of the dimer geometry on the entropy that are not reflected in simpler theories are identified. Specifically, for dimers adsorbing on the DNA double helix, the dimer geometry gives a persistently nonzero entropy and there is a significant charge inversion as the force binding the particles to the lattice increases relative to the thermal energy, which is not true of the simple lattice gas model for the dimers, in which all the sites are independent.

## 1. Introduction

From metabolism to drug design, the free energy of a system controls its biological processes. The free-energy minimum determines the equilibrium state. The free energy gradients drive the kinetics of the biochemical reactions.

The free energy contains both energy and entropy terms. The energy of complex biomolecules can now be calculated quite accurately, especially using the quantum–chemical methods introduced by Hohenberg and Kohn [1] and extensively developed by many authors in subsequent work. The entropy, on the other hand, is less-extensively studied, although the free energy involves the difference between the energy and entropy terms. The entropy term is known to play an important role in at least some biochemical processes. An example of an entropy-driven biochemical reaction can be found in sickle-cell disease, where the carbon dioxide causes hemoglobin polymerization and oxygen from respiration reverses the process [2,3]. In addition, strategies for controlling the entropy are being used in self-assembling systems to generate novel materials in fields like colloids, macromolecular systems and nonequilibrium assembly [4].

Biomolecules are typically long-chain molecules. Arrays of one-dimensional chains on a surface can be created by numerical methods like self-avoiding walks, as can two-dimensional self-avoiding membranes [See Chapter 10 of Plishke and Bergersen, [5]]. Analytic results, however, are few. The simplest “long-chain” molecule is the dimer, and the first analytic derivations of the partition function of such a system were by Temperley, Fisher, and Kasteleyn [6,7,8] for the classical dimer model. The simplest closed form expression for the partition function of a one-dimensional chain appears in Fisher’s 1961 paper [7], and we will be presenting a simplified version of their derivation. In the dimer model, when a dimer attaches to a lattice, it blocks two adjacent sites from other dimers attaching. It is much more difficult to determine the number of possible arrangement of dimers on a lattice than for only monomers, because a monomer only blocks a single site.

Fisher’s solution method for the dimer model builds monomers as well as dimers into the initial formal construction of the partition function. The monomer term complicates the derivation. Although one set of anticommuting matrices, similar to the Dirac matrices $\gamma^{\mu }$ of relativistic quantum mechanics, is used to enforce the dimer constraint, the presence of the monomer term requires an additional set of anticommuting matrices in a product form, reminiscent of the chirality operator $\gamma^{5}$, to create homogeneity. Even with this construction, Fisher was unable to find an analytic solution to complete the evaluation of the partition function on a two-dimensional lattice with both monomers and dimers included. He therefore dropped the monomer term to reach their final analytic expression for the partition function *Z* in dimension d=2.

Because of this, we present, beginning in Section 4, a simpler method of solving Fisher’s dimer model by neglecting the monomer term from the beginning, and hence restricting the monomer distribution on the simple square d=2 lattice to be completely filled with dimers, which we refer to as filling fraction ν=1, the completely filled lattice with no empty sites. We then need only a single set of anticommuting operators $A^{\mu }$ that live on the links of the lattice and that we refer to as link fields. This method also gives Fisher’s final analytic expression for the partition function *Z* in d=2. This result *Z* is for a finite lattice with the sites arranged in $n_{r}$ rows and $n_{c}$ columns.

Beginning in Section 8, we specifically use this result for the special case of a two-leg-ladder lattice with $n_{r}=2$ rows and $n_{c}\to \infty$ columns to construct, in the end, the partition function for an infinitely long one dimensional chain of sites containing not just dimers but also monomers of two colors. We use this to produce results using the dimer model that parallel the results found in previous papers for the DNA double helix [9,10], which use a lattice gas model. We find results from the dimer model that differ from the simpler lattice gas models, in which all the sites are independent. Perhaps the most important of these is that the dimer geometry gives a persistently nonzero entropy, as the force binding the particles to the lattice increases relative to the thermal energy.

## 2. Partition Function, Entropy, and Occupancy

Although the classical dimer problem only requires classical physics, classical statistical mechanics is plagued with oddities like Gibbs’s paradox. This means that it is more reliable to start from the quantum-mechanical expression for the partition function of the grand canonical ensemble,

(1) $$Z\equiv e^{-\beta \Omega_{G}}=\mathrm{Tr}\left(e^{-\beta (H-\sum_{j}\mu_{j}N_{j}}\right),$$

where $\Omega_{G}$ is the free energy of this ensemble (often called the grand canonical potential), *H* is the quantum Hamiltonian of the system, *N* is the number of particles in the system and $\mu_{j}$ the chemical potential of the particle of type *j*. The thermal parameter $\beta \equiv \frac{1}{k_{B}T}$ specifies the temperature *T* of the system, where $k_{B}$ is Boltzmann’s constant. The trace here is over quantum states of the system. Taking the trace is equivalent to summing over all the possible configurations of the system.

The entropy is then given by $S=-{\left(\frac{\partial \Omega_{G}}{\partial T}\right)}_{V,\{\mu_{j}\}}$, and the mean number of particles of type *j* is given by $\langle N_{j}\rangle =-{\left(\frac{\partial \Omega_{G}}{\partial \mu_{j}}\right)}_{T,V,\{\mu_{k\neq j}\}}$, where the subscripts indicate the quantities that are held constant in taking the partial derivatives.

Fisher’s dimer model describes dimers adsorbed on a two-dimensional lattice. Eventually, we will take the limit in which the lattice becomes large, and for that reason, it is useful to express the quantities as energy per lattice site and entropy per lattice site. Therefore, it is more reasonable to consider the entropy per site and the number of particles of a given type per site, which is the occupancy per site. It is then helpful to define the logarithm of partition function per site as

(2) $$\ln Z_{\mathrm{site}}=\lim_{N\to \infty }\frac{1}{N}\ln Z,$$

which means that the entropy per site can be written as

(3) $$S_{\mathrm{site}}=\lim_{N\to \infty }\frac{1}{N}\frac{\partial }{\partial T}{\left(\frac{1}{\beta }\ln Z\right)}_{V,\{\mu_{\gamma }\}}=\frac{\partial }{\partial T}\left(\frac{1}{\beta }\ln Z_{\mathrm{site}}\right)=-k_{B}\beta^{2}\frac{\partial }{\partial \beta }\left(\frac{1}{\beta }\ln Z_{\mathrm{site}}\right).$$

The derivative with respect to β gives

(4) $$\frac{\partial }{\partial \beta }\left(\frac{1}{\beta }\ln Z_{\mathrm{site}}\right)=-\frac{1}{\beta^{2}}\ln Z_{\mathrm{site}}+\frac{1}{\beta Z_{\mathrm{site}}}\frac{\partial Z_{\mathrm{site}}}{\partial \beta },$$

and then the entropy becomes

(5) $$S_{\mathrm{site}}=k_{B}\ln Z_{\mathrm{site}}-\frac{k_{B}\beta }{Z_{\mathrm{site}}}\frac{\partial Z_{\mathrm{site}}}{\partial \beta }.$$

A simple example of partition function is that of a lattice gas. Suppose that there are two kinds of particles, like red balls and blue balls. There are $(N_{r}+N_{b})!=N!$ ways to fill the *N* sites of the lattice, where $N_{r}$ and $N_{b}$ are the numbers of red and blue balls. This N! is divided by $N_{r}!$, the number of ways to swap red balls, and still has the same configuration, then by $N_{b}!$, the number of ways to swap blue balls without changing the configuration. This gives the number of configurations of $N_{r}$ red balls and $N_{b}$ blue balls,

(6) $$g_{N}\left(N_{r},N_{b}\right)=\frac{N!}{N_{b}!N_{r}!}.$$

There are no forces between the balls, merely a force holding them on the lattice sites, and so the Hamiltonian is

(7) $$H=\varepsilon_{r}N_{r}+\varepsilon_{b}N_{b},$$

while the chemical potential term becomes $\mu_{r}N_{r}+\mu_{b}N_{b}$. The partition function is then

(8) $$Z=\mathrm{Tr}(e^{-\beta (\varepsilon_{r}N_{r}+\varepsilon_{b}N_{b}-\mu_{r}N_{r}-\mu_{b}N_{b})}).$$

The activity of the red balls is $r=e^{-\beta (\varepsilon_{r}-\mu_{r})}$ and of the blue balls is $b=e^{-\beta (\varepsilon_{b}-\mu_{b})}$. If $g_{N}\left(N_{r},\;N_{b}\right)$ is the number of states in the trace that contain $N_{r}$ red balls and $N_{b}$ blue balls, then the partition function for this system becomes

(9) $$Z=\sum_{N_{r},N_{b}}g\left(N_{r},\;N_{b}\right)r^{N_{r}}b^{N_{b}}=\sum_{N_{r},N_{b}}\frac{N!}{N_{r}!\left(N-N_{r}\right)!}r^{N_{r}}b^{N_{b}},$$

where $N=N_{r}+N_{b}$. With the binomial theorem, this can be written as

(10) $$Z={\left(r+b\right)}^{N},$$

which is the partition function for red and blue balls on a lattice. The sites are independent, so that the factor r+b is the partition function contribution from each site. For an infinite lattice, the logarithm of the partition function per site is then

(11) $$\ln Z_{\mathrm{site}}=\lim_{N\to \infty }\frac{1}{N}\ln{\left(r+b\right)}^{N}=\ln\left(r+b\right).$$

The entropy per site is then given by

(12) $$S_{\mathrm{site}}=k_{B}\ln\left(r+b\right)-\frac{k_{B}\beta }{r+b}\frac{\partial (r+b)}{\partial \beta }.$$

Since β is contained in the activities *r* and *b*, $\frac{\partial r}{\partial \beta }=-\left(\varepsilon_{r}-\mu_{r}\right)r=\frac{1}{\beta }r\ln r$, and similarly for *b*. Inserting the explicit form of the partition function yields the entropy per site of the two-color lattice gas,

(13) $$S_{\mathrm{site}}=-k_{B}\ln\left(r+b\right)+\frac{k_{B}\beta }{\left(r+b\right)}\left(r\ln r+b\ln b\right).$$

We can similarly find the mean particle numbers by differentiating by the chemical potentials. For the red balls *r*,

(14) $$\langle N_{r}\rangle =\frac{1}{\beta }\frac{\partial \ln Z}{\partial \mu_{r}}.$$

The number of red balls per site $\langle n_{r}\rangle$ is given by

(15) $$\langle n_{r}\rangle =\lim_{N\to \infty }\frac{\langle N_{r}\rangle }{N}=\frac{1}{\beta }\frac{\partial }{\partial \mu_{r}}\ln Z_{\mathrm{site}}=\frac{1}{\beta Z_{\mathrm{site}}}\frac{\partial Z_{\mathrm{site}}}{\partial \mu_{r}}.$$

Substituting the partition function per site for the red balls, we have

(16) $$\langle n_{r}\rangle =\frac{1}{\beta (r+b)}\frac{\partial (r+b)}{\partial \mu_{r}}.$$

The derivative of *r* with respect to $\mu_{r}$ is given by $\frac{\partial r}{\partial \mu_{r}}=\beta r$, and so the average occupation of the red balls is

(17) $$\langle n_{r}\rangle =\frac{r}{r+b}.$$

Similarly, for blue balls, we have

(18) $$\langle n_{b}\rangle =\frac{b}{r+b},$$

and these two expressions add to one, which they should.

Generalizing this argument to include red, blue, and green balls, we have

(19) $$Z={\left(r+b+g\right)}^{N},$$

with the partition function per site in the infinite lattice limit represented as

(20) $$\ln Z_{\mathrm{site}}=\ln\left(r+b+g\right),$$

the entropy per site as

(21) $$S_{\mathrm{site}}=-k_{B}\ln\left(r+b\right)+\frac{k_{B}\beta }{\left(r+b\right)}\left(r\ln r+b\ln b+g\ln g\right),$$

and the occupancies as

(22) $$\langle n_{r}\rangle =\frac{r}{r+b+g},\;\langle n_{b}\rangle =\frac{b}{r+b+g},\;\langle n_{g}\rangle =\frac{g}{r+b+g}.$$

If one simply wants vacant sites rather than the green balls or holes in the lattice, the activity is replaced by $g=e^{-\beta (\varepsilon_{g}-\mu_{g})}=1$, because holes have neither an energy nor a chemical potential.

## 3. The Lattice and Its Dual

Dimer models describe the behavior of rods with ends that occupy sites on a lattice. However, two dimers cannot have their ends on the same site, and this is the fundamental constraint that makes determination of the allowed dimer configurations difficult. A physical example of a dimer might be a hydrogen molecule, with each of the two atoms held to a separate lattice site through electrostatic attraction. The lattice may be completely covered with dimers, or the coverage may be less than complete. In the latter case, the empty sites may be called vacancies, or alternatively regarded as occupied by monomers. The lattice can be of any dimension, although pictures of one- and two-dimensional lattices are easiest to draw.

Dimer models can be either classical or quantum. The difference is, basically, that in the quantum dimer model, the dimers can tunnel between sites, while classically, they can only move by thermally activated diffusive processes. The quantum dimer model is the basis for Anderson’s resonating-valence-bond theory of antiferromagnetism.

Fisher [7] considered a simple square lattice in two dimensions, with dimers placed randomly on this lattice. He then attempted to determine the number of different dimer arrangements. This result could, for example, be used to determine the entropy, because entropy is the logarithm of the number of possible states of a system, Fisher discovered that he could solve this problem when the lattice was fully occupied and there were no vacancies. The key, he found, was to randomly distribute “half dimers” on all sites of the lattice, as in the lattice gas problem. He then threw away all arrangements in which a half dimer was not connected to a neighbor because the orientations did not match. The remaining configurations were those in which dimers completely covered the lattice.

The trick he used to accomplish this feat was to tag each dimer with a member of a sufficiently large set of anticommuting objects $A_{j}$ such that

(23) $${\left[A_{i},\;A_{j}\right]}_{+}=A_{i}A_{j}+A_{j}A_{i}=2\delta_{ij},$$

so that $A_{j}^{2}=I$, with *I* the identity, where the $A_{j}$s were represented by matrices. He then used the trace theorems that are employed in particle physics to simplify calculations involving products of Dirac matrices [11,12,13]. These caused the unwanted configurations to vanish, and he identified those that remained with a Pfaffian. Its evaluation gives the number of dimer configurations.

When the lattice is completely covered with dimers, the calculation simplifies because there are no monomers. The fraction of sites covered by dimers is the “filling fraction”, ν, and this complete dimer coverage corresponds to ν=1.

The trace theorems evaluate products of the $A_{i}$s. We will need four different $A_{i}$s for each site in the lattice, and for all of them to be independent matrices, their dimension *d* will be quite large. Fortunately, all we need is the commutation law, because it defines their algebra. We will never have to actually write down any of these matrices.

**Figure 1:**
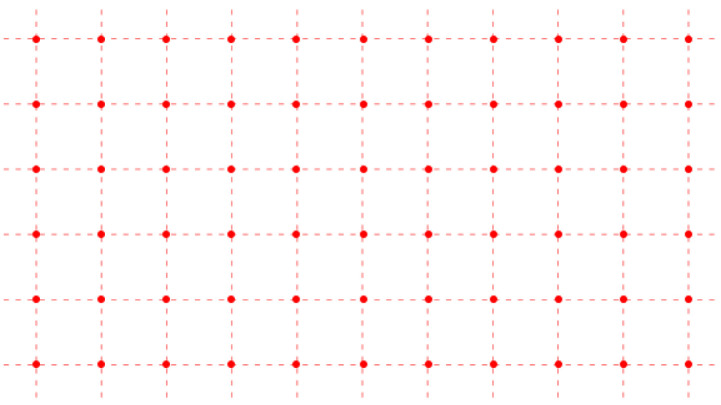
A simple square lattice with lattice points represented by red dots and the links represented by red dashed lines.

**Figure 2:**
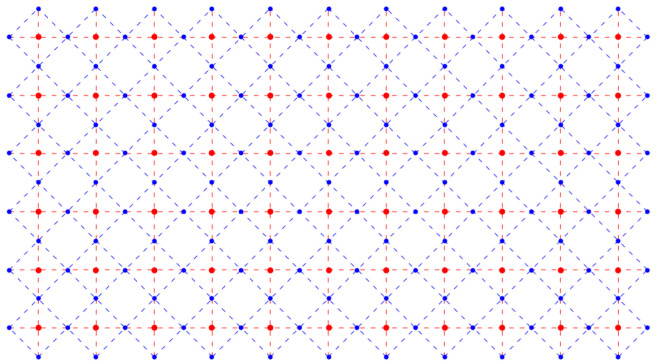
A simple square lattice with lattice points represented by red dots and the links represented by red dashed lines. The blue dots label dual lattice points, and the links of the dual lattice are blue dashed lines.

We consider dimers adsorbed on a simple square lattice of sites, indicated by the red points in Figure 1. The dual lattice is formed by connecting the midpoints of the links of the original lattice. The links are the lines joining each pair of neighboring sites (red, dashed). The dual lattice is the lattice of blue points located at the midpoints of the links of the original red lattice shown in Figure 2.

Let us denote the lattice sites by Roman subscripts i, j,⋯. The anticommuting objects A actually live on the links of the lattice, or equivalently on the sites of the dual lattice. Because they live on the links, one can think of them as "link fields" that anticommute. Let us use Greek superscripts α, β, γ, ⋯ to indicate the links or sites of the dual lattice, so that these anticommuting link fields can be written as $A^{\mu }$. These link fields are regarded here as operators that act on some set of vectors |α〉 in a vector space. The introduction of a representation by using a set of basis functions allows these fields $A^{\mu }$ to be represented by matrices of some suitable dimension *d*. The trace is then the sum of the diagonal elements $\langle \alpha |A^{\mu }|\alpha \rangle$, which is invariant under a unitary transformation.

The next step is to develop the trace theorems involving the fields $A^{\mu }$ that are needed here. Because Kronecker deltas and identity matrices are so easy to sum out, it is easy to lose track of them. It is clearer to write the anticommutation law as

(24) $${\left[A^{\mu },\;A^{\nu }\right]}_{+}=A^{\mu }A^{\nu }+A^{\nu }A^{\mu }=2g^{\mu \nu },$$

where $g^{\mu \nu }\equiv \delta^{\mu \nu }$, the Euclidean-space metric. When matrices of dimension *d* are used to represent the $A^{\mu }$, this becomes $g^{\mu \nu }\equiv \delta^{\mu \nu }I_{d}$ with $I_{d}$ the *d*-dimensional identity matrix. We will need to evaluate expressions involving $\frac{1}{d}\mathrm{Tr}\left(\cdots \right)$, where Tr(⋯) means the trace over a product of matrices representing the $A^{\mu }$.

A proof of the trace theorems of these matrices is given in Appendix A. A simpler way to look at the procedure is as follows. In the general product $A^{\mu_{1}}A^{\mu_{2}}\cdots A^{\mu_{n}}$, use anticommutation to move $A^{\mu }$s with the same index next to one another as pairs. This introduces a parity factor ${\left(-1\right)}^{P}$. Then, replace each matched pair with $I_{d}$, since the anticommutator ${\left[A^{\mu },\;A^{\nu }\right]}_{+}=2g^{\mu \nu }$ gives ${\left(A^{\mu }\right)}^{2}=g^{\mu \mu }=I_{d}$. You will obtain $\mathrm{Tr}(I_{d})=d$ if the product consists only of matched pairs. Otherwise, there will be at least one $g^{\mu \nu }$ factor that has unmatched indices, and the entire product will vanish. The trace theorems for the $A^{\mu }$ are as follows:

*3.1*  *If the general product $A^{\mu_{1}}A^{\mu_{2}}\cdots A^{\mu_{n}}$ can be rearranged so that adjacent pairs of indices are the same, then*
  (25) $$\frac{1}{d}\mathrm{Tr}\left(A^{\mu_{1}}A^{\mu_{2}}\cdots A^{\mu_{n}}\right)={\left(-1\right)}^{P},$$
  *where P is the appropriate parity index for the rearrangement.*
*3.2*  *If the general product $A^{\mu_{1}}A^{\mu_{2}}\cdots A^{\mu_{n}}$ cannot be rearranged so that adjacent pairs of indices are the same, then*
  (26) $$\mathrm{Tr}(A^{\mu_{1}}A^{\mu_{2}}\cdots A^{\mu_{n}})=0.$$

## 4. The Dimer Model

**Figure 3:**
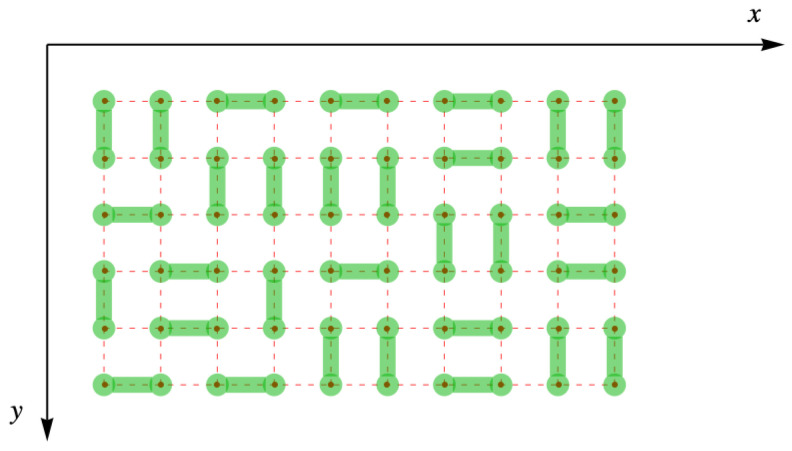
Complete filling of dimers (green) on the simple square lattice (red dots and dashes).

One of the simplest models of a system containing diatomic molecules is that of lattice gas of $N_{d}$ rigid dimers, each of which fills two nearest neighbor sites of a space lattice of $N_{\mathrm{sites}}$ sites. Fisher was only able to completely evaluate their result for a lattice completely filled, ν=1, with dimers, leaving no vacancies or monomers, as shown in Figure 3. Furthermore, because we will eventually represent the lattice with its dimer arrangements by matrices, the y-axis is inverted so that the site (x=1, y=1) is in the upper left corner like the usual initial matrix element $M_{11}$.

We suppose that the dimers do not interact with one another apart from the geometric constraint that only one dimer can be attached to a given site. The dimers are bound to the lattice sites, and we let the total binding energy of a dimer be ε, which is twice the binding energy to each site because a dimer has two ends. We follow Fisher [7] in allowing the binding energy of dimers aligned in the two orthogonal directions to be different, and call them $\varepsilon_{x}$ and $\varepsilon_{y}$. Then, the partition function becomes

(27) $$Z=\mathrm{Tr}(e^{-\beta [\left(\varepsilon_{x}-\mu_{x}\right)N_{x}+\left(\varepsilon_{y}-\mu_{y}\right)N_{y}]}).$$

Here, $x\equiv e^{-\beta (\varepsilon_{x}-\mu_{x})}$ is the activity of an *x*-oriented dimer, and we also let $y\equiv e^{-\beta (\varepsilon_{y}-\mu_{y})}$ be the activity of a *y*-oriented dimer, following Fisher, who allowed the dimer activities in the two directions to differ. The trace over quantum states adds together the contributions of states with the same numbers $N_{x}$ and $N_{y}$ of dimers oriented in the two directions. If we define the number of such states to be $g(N_{x},\;N_{y})$, an equivalent expression for the partition function is

(28) $$Z=\sum_{N_{x},N_{y}}g\left(N_{x},\;N_{y}\right)x^{N_{x}}y^{N_{y}}.$$

which is actually valid for any filling fraction ν.

**Figure 4:**
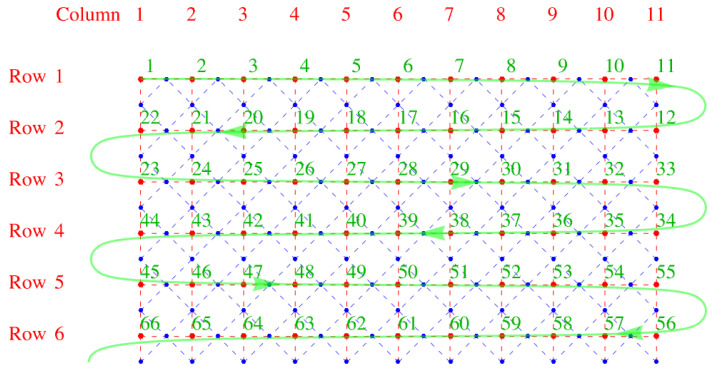
Serpentine numbering of the lattice sites in green. The row and column numbers of the lattice are also shown The red dots and dashes outline the direct lattice, and the blue dots and dashes the dual lattice.

We now invent, following Fisher, a snake-like path through the lattice that allows us to use a one-dimensional numbering scheme to label the lattice sites. The numbering begins at the upper left corner and weaves back and forth along the *x*-direction, as shown in Figure 4. We note that this one-dimensional numbering scheme, serpentine numbering, also works for a three-dimensional lattice if one draws it on a long sheet of paper and then folds the paper with accordion-like pleats. For a lattice with $n_{r}$ rows of sites (red) arranged one below another in the vertical *y*-direction and $n_{c}$ columns parallel to one another in the horizontal *x*-direction, we have a lattice of $N_{\mathrm{sites}}=n_{r}n_{c}$ sites. The virtue of this numbering is that it is easy to determine the signs resulting from the interchanges required to move identical $A^{\mu }$s adjacent to one another. For the *x*-links it is obvious, because the two ends of a dimer are on adjacent sites, so no interchange is needed, and the sign is plus. For a *y*-link, consider the following example: the link between sites 27 and 40 in Figure 4. The *y*-links (vertical red dashed lines) look like the ties between two rails on a railroad track, and these ties have two ends. Thus, to move $A^{\mu }$ from position 27 to 39 (adjacent to 40) requires moving to the right by six interchanges (27→28→29→30→31→32→33) along row three, and then moving back left along row 4 by another six interchanges (33→34→35→36→37→38→39). Because a tie has two ends, you will always obtain an even number of interchanges, and this will crucially make all terms positive in our construction of the partition function below.

The dual lattice has twice as many sites as the original lattice because there are two links per site in the square lattice. Consequently, if the original lattice has $N_{\mathrm{sites}}$ sites, the dual lattice has $2N_{\mathrm{sites}}$ links or points. However, there are only $\frac{1}{2}N_{\mathrm{sites}}$ distinct adjacent pairs of points in the original lattice, and ν=1 means that all of these $\frac{1}{2}N_{\mathrm{sites}}$ distinct adjacent pairs of points are occupied by dimers. This makes it generally useful to consider $N_{\mathrm{sites}}$ as even, because if it is odd, there is at least one vacancy.

**Figure 5:**
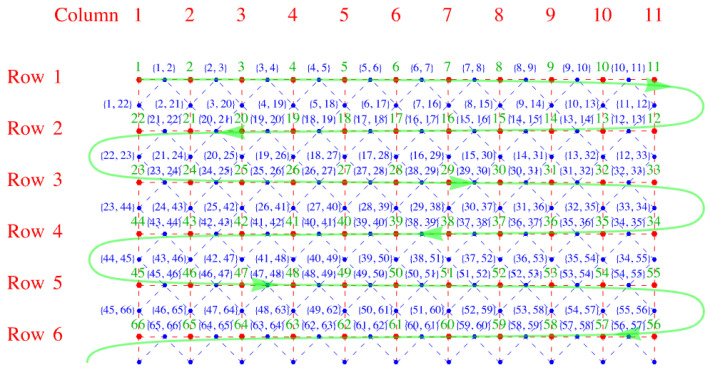
Alternative way of numbering of links of the lattice by using pairs of site indices, shown in blue, on top of Figure 4.

An alternative way of labeling the links is to make the superscript μ of the link field $A^{\mu }$ a pair of site indices μ=(j, k) where *j* and *k* label the two ends of the link, where j<k. This leads to the link-field labels of Figure 5.

**Figure 6:**
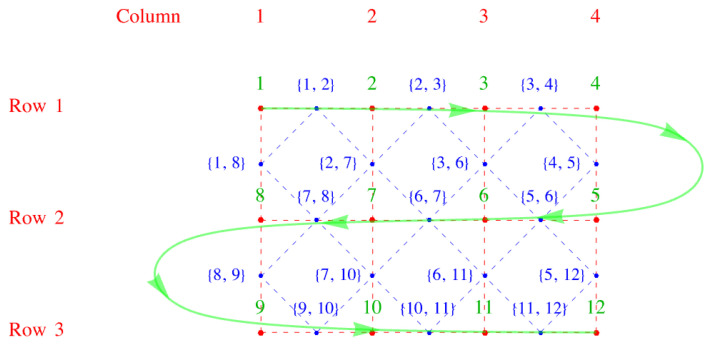
A smaller 12×12 example with four rows and three columns, but similar to Figure 5, where the serpentine numbering is shown in green, and the alternative numbering in blue. As before, the red dots and dashes outline the direct lattice, and the blue dots and dashes the dual lattice.

The link fields can be arranged as the upper right triangle of an $N_{\mathrm{sites}}\times N_{\mathrm{sites}}$ matrix with zeros down the diagonal, suggestive of an antisymmetric matrix. For a smaller 12×12 example with four rows and three columns but similar to the example above, the upper right triangle of entries $A^{jk}$ is shown in Equation (29) and shown pictorially in Figure 6.

(29) $$A^{jk}=\left(\begin{array}{cccccccccccc} 0 & A^{1,2} & 0 & 0 & 0 & 0 & 0 & A^{1,8} & 0 & 0 & 0 & 0\\ & 0 & A^{2,3} & 0 & 0 & 0 & A^{2,7} & 0 & 0 & 0 & 0 & 0\\ & & 0 & A^{3,4} & 0 & A^{3,6} & 0 & 0 & 0 & 0 & 0 & 0\\ & & & 0 & A^{4,5} & 0 & 0 & 0 & 0 & 0 & 0 & 0\\ & & & & 0 & A^{5,6} & 0 & 0 & 0 & 0 & 0 & A^{5,12}\\ & & & & & 0 & A^{6,7} & 0 & 0 & 0 & A^{6,11} & 0\\ & & & & & & 0 & A^{7.8} & 0 & A^{7,10} & 0 & 0\\ & & & & & & & 0 & A^{8,9} & 0 & 0 & 0\\ & & & & & & & & 0 & A^{9,10} & 0 & 0\\ & & & & & & & & & 0 & A^{10,11} & 0\\ & & & & & & & & & & 0 & A^{11,12}\\ & & & & & & & & & & & 0 \end{array}\right).$$

The link fields $A^{1,2}$, $A^{2,3}$, and $A^{3,4}$ refer to *x*-directed links, and the link fields $A^{4,5}$, $A^{3,6}$, $A^{2,7}$, and $A^{1,8}$ refer to *y*-directed links. There are no near-neighbor links corresponding to the elements for which zero is entered. In other words, the entries sloping diagonally upward in the direction lower left corner to upper right corner are *y*-directed links. The remaining ones sloping downward from the upper left to lower right corners are *x*-directed links in the pattern shown in the matrix *M* in Equation (30).

(30) $$M=\left(\begin{array}{cccccccccccc} 0 & x & 0 & 0 & 0 & 0 & 0 & y & 0 & 0 & 0 & 0\\ & 0 & x & 0 & 0 & 0 & y & 0 & 0 & 0 & 0 & 0\\ & & 0 & x & 0 & y & 0 & 0 & 0 & 0 & 0 & 0\\ & & & 0 & y & 0 & 0 & 0 & 0 & 0 & 0 & 0\\ & & & & 0 & x & 0 & 0 & 0 & 0 & 0 & y\\ & & & & & 0 & x & 0 & 0 & 0 & y & 0\\ & & & & & & 0 & x & 0 & y & 0 & 0\\ & & & & & & & 0 & y & 0 & 0 & 0\\ & & & & & & & & 0 & x & 0 & 0\\ & & & & & & & & & 0 & x & 0\\ & & & & & & & & & & 0 & x\\ & & & & & & & & & & & 0 \end{array}\right).$$

## 5. Inclusion of Constraints on the Partition Function by Use of a Child’s Toy

**Figure 7:**
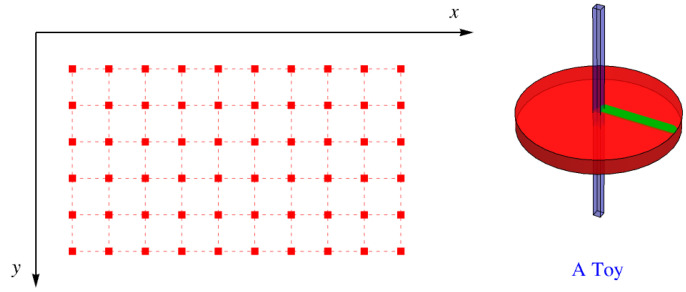
Square holes in a simple square lattice with a toy with a square shaft that can fit into a hole in one of four directions.

Suppose that the lattice has square holes with sides parallel to *x* and *y* at each lattice site. Into each of these holes, we insert a toy, like a child’s top with a square shaft that just fits the hole, as shown in Figure 7. The disk that provides most of the top’s angular momentum has a green line painted on it normal to one of the faces of the shaft. This means that the green line can be oriented four ways—north, south, east, and west.

**Figure 8:**
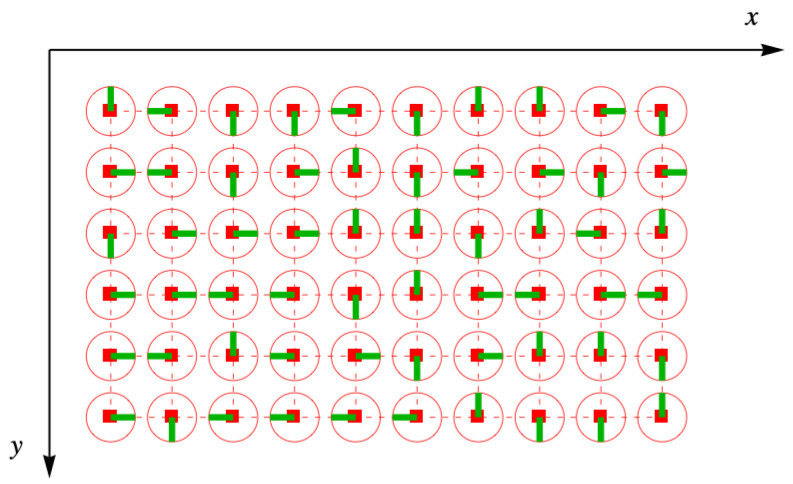
Toys randomly arranged on the simple square lattice, shown as the red square holes and connecting dashes. The half-dimers are represented by the green lines for this particular member of the ensemble, with the red circles the outlines of the toys in Figure 7. Not all the half-dimers point toward each other in pairs, and so taking the trace over link fields will cause this distribution to be removed from the ensemble.

The orientations of the disk are random, so they are distributed like a lattice gas of four colors, as shown in Figure 8. Sometimes, the green lines of neighboring disks point toward one another, and for any adjacent pair, the probability of this occurring is 1/(4×4)=1/16.

**Figure 9:**
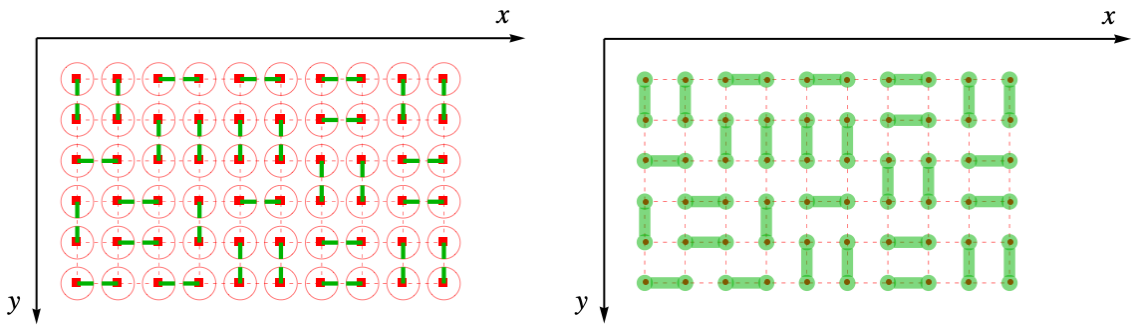
Half-dimers arranged on the simple square lattice. In the left figure, the dimers all point toward one another in pairs, so that taking the trace over link fields will retain this as a member of the ensemble, unlike the random arrangement of Figure 8. This corresponds to the completely filled dimer arrangement on the right, which is the same as in Figure 3.

Now, suppose that the green lined represent “half-dimers”, and when green lines point toward one another on adjacent sites, those two half-dimers join up to form a complete dimer between those two sites. In Figure 9, the figure on the left shows the half-dimers pointing toward one another on adjacent sites, and the figure on the right shows the corresponding dimer arrangement.

We construct the partition function in a way that allows the constraints of no double occupancy and a ν=1 completely filled dimer arrangement to be imposed. Associate with each lattice site *j* a function

(31) $$V_{j}=\sum_{\begin{array}{c} l=1\\ nn \end{array}}\sqrt{z_{jl}}\;A^{jl},$$

where the sum is over the nearest neighbors of site *j*, as emphasized by the subscript nn on the sum. The quantity $z_{jl}$ is the activity of a dimer on the link jl, and is either $x_{jl}$ if the link is in the *x*-direction, or $y_{jl}$ if the link is in the *y*-direction. The factor $A^{jk}$ is the link field associated with the link jl. The square root of the activity is taken because it is the activity of a half-dimer, the object represented by the green line on the disk of the toy. It takes two of these factors, ${\left(\sqrt{z_{jl}}\right)}^{2}=z_{jl}$, to give the activity of the dimer on the link. One factor can be thought of as emanating from the site *j*, and the other from the site *ℓ*.

The partition function is constructed as a product of these factors $V_{j}$, one rooted on each lattice site *j*. To see how this will work, suppose that we have a product of only two lattice sites called *j* and *k*. The product is

(32) $$\begin{array}{ccc} V_{j}V_{k} & = & \left(\sum_{\begin{array}{c} l=1\\ nn \end{array}}^{4}\sqrt{z_{jl}}\;A^{jl}\right)\left(\sum_{\begin{array}{c} m=1\\ nn \end{array}}^{4}\sqrt{z_{km}}\;A^{km}\right)\\ & = & \left(\sqrt{x_{jl_{1}}}\;A^{jl_{1}}+\sqrt{y_{jl_{2}}}\;A^{jl_{2}}+\sqrt{x_{jl_{3}}}\;A^{jl_{3}}+\sqrt{x_{jl_{4}}}\;A^{jl_{4}}\right)\times \\ & & \times \left(\sqrt{x_{km_{1}}}\;A^{km_{1}}+\sqrt{y_{km_{2}}}\;A^{km_{2}}+\sqrt{x_{km_{3}}}\;A^{km_{3}}+\sqrt{y_{km_{4}}}\;A^{km_{4}}\right)\\ & = & \sqrt{x_{jl_{1}}}\sqrt{k_{km_{1}}}A^{jl_{1}}A^{km_{1}}+\sqrt{x_{jl_{1}}}\sqrt{y_{km_{2}}}A^{jl_{1}}A^{km_{2}}\\ & & +\sqrt{x_{jl_{1}}}\sqrt{x_{km_{3}}}A^{jl_{1}}A^{km_{3}}+\sqrt{x_{jl_{1}}}\sqrt{y_{km_{4}}}A^{jl_{1}}A^{km_{4}}+\cdots . \end{array}$$

If sites *j* and *k* are not nearest neighbors, then none of the link fields are common to both factors, and taking $\frac{1}{d}Tr\cdots$ simply gives zero and the term vanishes. None of the sixteen terms in the last line then will contribute to the partition function.

The situation is different when *j* and *k* are nearest neighbors. Then, there will be one link field in common. Suppose, for example, that it is on an *x*-directed link, arising from

(33) $$V_{j}V_{k}=\cdots +\sqrt{x_{jl_{1}}}\sqrt{x_{km_{3}}}A^{jl_{1}}A^{km_{3}}+\cdots =\cdots +\sqrt{x_{jk}}\sqrt{x_{kj}}A^{jk}A^{kj}+\cdots ,$$

because *j* and *k* are nearest neighbors in the *x*-direction with *k* to the right of *j*. Since the order of subscripts on a link does not matter, $\sqrt{x_{kj}}\sqrt{x_{kj}}={\left(\sqrt{x_{kj}}\right)}^{2}=x^{2}$. Thus, this term survives when the trace is taken.

In this way, the partition function,

(34) $$Z=\frac{1}{d}\mathrm{Tr}\prod_{j=1}^{N_{\mathrm{sites}}}V_{j}=\frac{1}{d}\mathrm{Tr}\;V_{1}V_{2}\cdots V_{N_{\mathrm{sites}}},$$

is an expression in which the only surviving terms are those which give ν=1 dimer arrangements, as shown, for example, in Figure 9. The numbering is one-dimensional, $123\cdots N_{\mathrm{sites}}$, because we use one-dimensional serpentine ordering.

## 6. Introduction of the Antisymmetric Matrix

A more concise notation is useful. Furthermore, we want the contributions of the even-numbered and the odd-numbered sites to the partition function to be written slightly differently. We begin by writing

(35) $$V_{j}=\sum_{l=1}^{4}v_{jl}A^{jl},$$

so that $v_{jl}\equiv \sqrt{z_{jl}}$, which is, of course, either $\sqrt{x}$ or $\sqrt{y}$, and where the subscript “nn" on the sum is omitted, although the restriction of the sum in $V_{j}$ to nearest neighbors of *j* will remain understood.

Now, consider the product,

(36) $$\prod_{j=1}^{N_{\mathrm{sites}}}V_{j}=V_{1}V_{2}\cdots V_{N_{\mathrm{sites}}},$$

which appears in the partition function. The number of sites is chosen to be even, and we insert 1=(−i)i between each odd–even pair, giving

(37) $$\begin{array}{ccc} \prod_{j=1}^{N_{\mathrm{sites}}}V_{j} & = & V_{1}\left(-i\right)iV_{2}V_{3}\left(-i\right)iV_{4}\cdots V_{N_{\mathrm{sites}}-1}\left(-i\right)iV_{N_{\mathrm{sites}}}\\ & = & \left(-iV_{1}\right)\left(iV_{2}\right)\left(-iV_{3}\right)\left(iV_{4}\right)\cdots \left(-iV_{N_{\mathrm{sites}}-1}\right)(iV_{N_{\mathrm{sites}})}. \end{array}$$

Then, for odd-numbered sites, we have

(38) $$Q_{j=odd}\equiv \left(-i\right)V_{j=odd}=\sum_{l=1}^{4}\left(-i\right)v_{jl}A^{jl},$$

and for even-numbered sites, we have

(39) $$Q_{j=even}\equiv iV_{j=even}=\sum_{l=1}^{4}iv_{jl}A^{jl}.$$

These relations can be combined as

(40) $$Q_{j}=\sum_{l=1}^{4}{\left(-i\right)}^{j}iV_{jl}A^{jl},$$

and the original product becomes

(41) $$\prod_{j=1}^{N_{\mathrm{sites}}}V_{j}=\prod_{j=1}^{N_{\mathrm{sites}}}Q_{j}.$$

Finally, let us set

(42) $$Z=\frac{1}{d}\mathrm{Tr}\prod_{j=1}^{N_{\mathrm{sites}}}Q_{j}=\frac{1}{d}\mathrm{Tr}\;Q_{1}Q_{2}\cdots Q_{N_{\mathrm{sites}}}.$$

The quantities $Q_{j}$ are operators formed as linear combinations of the link fields. The link fields satisfy the simple anticommutation law

(43) $${\left[A^{\mu },\;A^{\nu }\right]}_{+}=A^{\mu }A^{\nu }+A^{\nu }A^{\mu }\equiv 2g^{\mu \nu }=2\delta^{\mu \nu },$$

or, for *d*-dimensional matrix representations thereof,

(44) $${\left[A^{\mu },\;A^{\nu }\right]}_{+}=A^{\mu }A^{\nu }+A^{\nu }A^{\mu }\equiv 2g^{\mu \nu }=2\delta^{\mu \nu }I_{d}.$$

We now show that the $Q_{j}$ operators also anticommute, and calculate the values of their anticommutators. We have

(45) $${\left[Q_{j},\;Q_{k}\right]}_{+}=Q_{j}Q_{k}+Q_{k}Q_{j}=\sum_{l=1}^{4}q_{jl}A^{jl}\sum_{m=1}^{4}q_{km}A^{km}+\sum_{m=1}^{4}q_{km}A^{km}\sum_{l=1}^{4}q_{jl}A^{jl}.$$

Writing this in terms of the anticommutators of the link fields gives

(46) $${\left[Q_{j},\;Q_{k}\right]}_{+}=\sum_{l=1}^{4}q_{jl}\sum_{m=1}^{4}q_{km}\left(A^{jl}A^{km}+A^{km}A^{jl}\right)=2\sum_{l=1}^{4}\sum_{m=1}^{4}q_{jl}q_{km}\delta^{\left(jl),(km\right)},$$

where $\delta^{\left(jl),(km\right)}$ gives unity if jl and km denote the same link field and zero otherwise. They only denote the same link field $A^{\mu }$ if the lattice sites *j* and *k* are nearest neighbors, and then only if m=j and l=k, that is, $\delta^{\left(jl),(km\right)}=\delta_{jm}\delta_{kl}$. The anticommutator becomes

(47) $${\left[Q_{j},\;Q_{k}\right]}_{+}=2\sum_{l=1}^{4}\sum_{m=1}^{4}q_{jl}q_{km}\delta_{jm}\delta_{kl},$$

which reduces to

(48) $${\left[Q_{j},\;Q_{k}\right]}_{+}=2q_{jk}q_{kj}.$$

Finally, we write this in terms of the notation $q_{jl}={\left(-1\right)}^{j}iv_{jl}$ of Section 5. This gives

(49) $${\left[Q_{j},\;Q_{k}\right]}_{+}=2v_{jk}v_{kj},$$

if *j* and *k* are nearest neighbors, and zero otherwise.

Furthermore, $v_{jl}=\sqrt{z_{jl}}$, the square root of the activity of a dimer on the link jl, which does not depend on the order of the indices. Thus, the anticommutator of the operators $Q_{j}$ and $Q_{k}$ can be written as

(50) $${\left[Q_{j},\;Q_{k}\right]}_{+}=2{\left(v_{jk}\right)}^{2}=2z_{jk},$$

the activity *x* or *y* of the dimer occupying that link.

The expression giving the partition function is

(51) $$Z=\frac{1}{d}\mathrm{Tr}\prod_{j=1}^{N_{\mathrm{sites}}}Q_{j}=\frac{1}{d}\mathrm{Tr}\;Q_{1}Q_{2}\cdots Q_{N_{\mathrm{sites}}}.$$

In order to have the lattice completely filled with dimers (filling factor ν=1), the number $N_{\mathrm{sites}}$ of lattice sites must be even in order to avoid a single essential but unwanted left over vacant site. As a reminder of this, let us temporarily set $N_{\mathrm{sites}}=2h$, and write the partition function as

(52) $$Z=\frac{1}{d}\mathrm{Tr}\;Q_{1}Q_{2}\cdots Q_{2h}.$$

To make further progress, we need to find a way to evaluate the product $\prod_{j=1}^{2h}Q_{j}$ that appears in the partition function

(53) $$Z=\frac{1}{d}\mathrm{Tr}\prod_{j=1}^{2h}Q_{j}.$$

To do this, we successively use the anticommutator of the operators $Q_{j}$ from Equation (50).

Let us write the anticommutator as

(54) $${\left[Q_{j},\;Q_{k}\right]}_{+}=2z_{jk}-Q_{k}Q_{j},$$

and use this expression to rearrange the product as

(55) $$\prod_{j=1}^{2h}Q_{j}=Q_{1}Q_{2}\cdots Q_{2h}=2z_{12}Q_{3}Q_{4}\cdots Q_{2h}+\left(-1\right)Q_{2}Q_{1}Q_{3}Q_{4}\cdots Q_{2h}.$$

The second term on the right has $Q_{1}$ displaced one step to the right, and can be written with the aid of the anticommutation rule as

(56) $$\begin{array}{ccc} \left(-1\right)Q_{2}Q_{1}Q_{3}Q_{4}\cdots Q_{2h} & = & \left(-1\right)Q_{2}\left(2z_{13}\right)Q_{4}\cdots Q_{2h}+{\left(-1\right)}^{2}Q_{2}Q_{3}Q_{1}Q_{4}\cdots Q_{2h}\\ & = & \left(-1\right)\left(2z_{13}\right)Q_{2}Q_{4}Q_{5}\cdots Q_{2h}+{\left(-1\right)}^{2}Q_{2}Q_{3}Q_{1}Q_{4}\cdots Q_{2h}. \end{array}$$

We continue to move $Q_{1}$ all the way to the right, requiring 2h−1 interchanges. We then take the trace of both sides, and use the cyclic property of the trace to move $Q_{1}$ back to the beginning, thereby reproducing the trace of the left side accompanied by ${\left(-1\right)}^{2h-1}=-1$. In the course of performing this, we “contract” $Q_{1}$ and $Q_{k}$ for k=2, 3, ⋯, 2h, producing 2h−1 terms containing a factor of $z_{1k}$, each accompanied by ${\left(-1\right)}^{k-1}$. This result is

(57) $$2\;\mathrm{Tr}\prod_{j=1}^{2h}Q_{j}=\sum_{k=2}^{2h}{\left(-1\right)}^{k-1}\left(2z_{1k}\right)\mathrm{Tr}\left[Q_{2}Q_{3}Q_{4}\cdots Q_{k-1}Q_{k+1}\cdots Q_{2h}\right].$$

The factors of two can cancel. Furthermore, $z_{1k}$ for k=2, 3, ⋯, 2h is the top row of a Pfaffian of order 2h.

Appendix B contains a brief description of Pfaffians and an example of how a simple Pfaffian is evaluated. On iterating, each step produces a single additional factor of $z_{kl}$ in every one of the terms resulting from the trace on the right. The end result is that the trace of Equation (57) produces the Pfaffian

(58) $$\mathrm{Tr}\prod_{j=1}^{2h}Q_{j}=\mathrm{Pf}\left[z_{jk}\right].$$

If *j* and *k* are extended to cover the entire lattice, the complete entries in the Pfaffian and the upper right triangle of the corresponding antisymmetric matrix will be filled in. In the present context, the only nonvanishing activities $z_{jk}$ are on links with *j* and *k* nearest-neighbor sites, and all the remaining entries are zero. This result is found in Fisher’s paper [14] and references therein, and is written in detail in J. C. Baker’s MS thesis [15].

Earlier, an example with three rows and four columns of sites was discussed, and is shown in Figure 6. The 12×12 antisymmetric matrix associated with the Pfaffian of this 3×4 lattice example, written in block form, is

(59) M(3×4)=0x00000y0000−x0x000y000000−x0x0y00000000−x0y0000000000−y0x00000y00−y0−x0x000y00−y000−x0x0y00−y00000−x0y0000000000−y0x00000000−y0−x0x000000−y000−x0x0000−y00000−x0.

and that block form consists of the nine 4×4 blocks, denoted as

**Figure 10:**
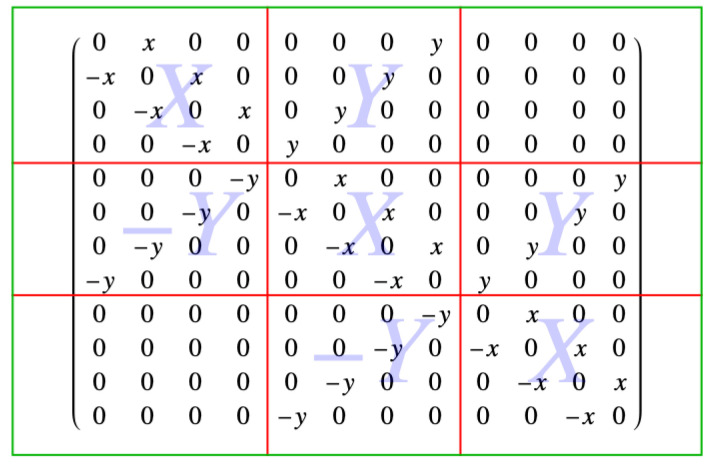
The block matrix form of the 12×12 antisymmetric matrix. The red lines outline the 4×4 blocks, and the light blue *X*, *Y* and −Y labels in the background signify the *X*, *Y* and −Y blocks of the matrix.

(60) M(3×4)=XY0−YXY0−YX,

as shown in Figure 10.

This is also a tridiagonal matrix, where the blocks *X*, *Y*, and 0 are 4×4 blocks. These blocks are

(61) $$X=\left(\begin{array}{cccc} 0 & x & 0 & 0\\ -x & 0 & x & 0\\ 0 & -x & 0 & x\\ 0 & 0 & -x & 0 \end{array}\right)$$

and

(62) $$Y=\left(\begin{array}{cccc} 0 & 0 & 0 & y\\ 0 & 0 & y & 0\\ 0 & y & 0 & 0\\ y & 0 & 0 & 0 \end{array}\right),$$

while “0” stands for a 4×4 block of sixteen zeros. In what follows, we will be using the fact that matrices can be multiplied block by block, and this is shown in Appendix C.

Consider the upper right triangle that forms the corresponding Pfaffian. The first row has $n_{c}-1$ *x*-oriented links between the $n_{c}$ sites, and so the block *X* has $n_{c}-1$ entries *x* in the direction of the diagonal. For the 3×4 example, there are four columns, so $n_{c}-1=3$ and there are three entries *x* in the block *X*. They are offset from the diagonal by one, so the dimensions of the block *X* shown are 4×4. In general, the blocks *X* are $\left(n_{c}-1+1\right)\times \left(n_{c}-1+1\right)=n_{c}\times n_{c}$.

Between each row and the one below it, there are $n_{c}$ vertical *y* links, and so there are $n_{c}$ entries of *y* up the diagonal line from the lower left to upper right corner. In the 3×4 example, $n_{c}=4$, and there are four of these so the block *Y* is 4×4. In general, the blocks *Y* are of dimension $n_{c}\times n_{c}$, just like the blocks *X*, as they must be to fill the original matrix of dimension $N_{\mathrm{sites}}\times N_{\mathrm{sites}}$, because $N_{\mathrm{sites}}=n_{r}n_{c}$. There are $n_{r}^{2}$ blocks with $n_{c}^{2}$ entries to account for the $N_{\mathrm{sites}}^{2}$ entries in the antisymmetric matrix *M*.

In general, then, when the lattice of sites has $n_{r}$ rows and $n_{c}$ columns, the dimension of each individual block is $n_{c}\times n_{c}$. The matrix of blocks, on the other hand, has dimension $n_{r}\times n_{r}$. The total number of matrix elements is then $n_{r}^{2}n_{c}^{2}$, which is the square of the number of sites $N_{\mathrm{sites}}=n_{r}n_{c}$.

## 7. Eigenvalues and Eigenvectors of the Asymmetric Matrix

The scheme that makes the calculation simplest is to diagonalize the *X* block and use those eigenvectors as the basis to transform the *Y* block. This will lead to a cruciform version of the big matrix, which will be convenient for the calculations. Therefore, first we investigate the eigenvalues and eigenvectors of the block *X*. The eigenvalue equation for *X* is given by

(63) Xv=λv,

where λ is an eigenvalue and *v* a column vector with entries $v_{1},\;v_{2},\;\cdots ,\;v_{n_{c}}$. The set of linear equations represented by this equation have the form

(64) $$\left(\begin{array}{ccccc} -\lambda & x & 0 & \cdots & 0\\ -x & -\lambda & x & \cdots & 0\\ \vdots & \ddots & \ddots & \ddots & \vdots \\ 0 & \cdots & -x & -\lambda & x\\ 0 & \cdots & 0 & -x & -\lambda \end{array}\right)\left(\begin{array}{c} v_{1}\\ v_{2}\\ v_{3}\\ \vdots \\ v_{n_{c}-1}\\ v_{n_{c}} \end{array}\right)=\left(\begin{array}{c} -\lambda v_{1}+xv_{2}\\ -xv_{1}-\lambda v_{2}+xv_{3}\\ \vdots \\ -xv_{n_{c}-2}-\lambda v_{n_{c}-1}+xv_{n_{c}}\\ -xv_{n_{c}-1}-\lambda v_{n_{c}} \end{array}\right)=\left(\begin{array}{c} 0\\ 0\\ \vdots \\ 0\\ 0 \end{array}\right).$$

We can make all the linear equations look the same if we imagine padding the list with $v_{0}$ and $v_{n_{c}+1}$ and then apply the boundary condition $v_{0}=v_{n_{c}+1}=0$. Then, we have

(65) $$\left(\begin{array}{c} -xv_{0}-\lambda v_{1}+xv_{2}\\ -xv_{1}-\lambda v_{2}+xv_{3}\\ \vdots \\ -xv_{n_{c}-2}-\lambda v_{n_{c}-1}+xv_{n_{c}}\\ -xv_{n_{c}-1}-\lambda v_{n_{c}}+xv_{n_{c}+1} \end{array}\right)=\left(\begin{array}{c} 0\\ 0\\ \vdots \\ 0\\ 0 \end{array}\right).$$

The generic equation is the difference equation

(66) $$-xv_{k-1}-\lambda v_{k}+xv_{k+1},$$

which can be rewritten as

(67) $$\frac{v_{k+1}-v_{k-1}}{2a}=\frac{\lambda }{2ax}v_{k}.$$

Regarding *a* as a lattice spacing, we recognize this equation as the discretized version of the first-order differential equation

(68) $$\frac{dv}{dt}=\frac{\lambda }{2ax}v\left(t\right),$$

with exponential solutions $v\left(t\right)=e^{\frac{\lambda t}{2ax}}$ that are growing or decaying if the eigenvalue λ is real. If λ is imaginary, the solutions oscillate.

A solution that satisfies the boundary conditions is given by

(69) $$v_{k}=A\left(e^{ik\varphi }-e^{i\pi k}e^{-ik\varphi }\right)=A\left[e^{ik\varphi }-{\left(-1\right)}^{k}e^{-ik\varphi }\right].$$

This gives sin(kφ) for *k* even but cos(kφ) for *k* odd.

The boundary condition at k=0 is

(70) $$v_{0}=A\left[1-{\left(-1\right)}^{0}1\right]=A\left(1-1\right)=0,$$

which is correct. At $k=n_{c}+1$ we have

(71) $$\begin{array}{ccc} v_{k=n_{c}+1} & = & A[e^{i(n_{c}+1)\varphi }-{\left(-1\right)}^{\left(n_{c}+1\right)}e^{-i(n_{c}+1)\varphi }]\\ & = & A[e^{i(n_{c}+1)\varphi }-{\left(e^{i\pi }\right)}^{\left(n_{c}+1\right)}e^{-i(n_{c}+1)\varphi }]\\ & = & A[e^{i(n_{c}+1)\varphi }-e^{-i\left(n_{c}+1\right)\left(\pi -\varphi \right)}]. \end{array}$$

We also require this to vanish, which occurs if the quantity in brackets vanishes, that is, if

(72) $$e^{i(n_{c}+1)\varphi }=e^{-i\left(n_{c}+1\right)\left(\pi -\varphi \right)}.$$

**Figure 11:**
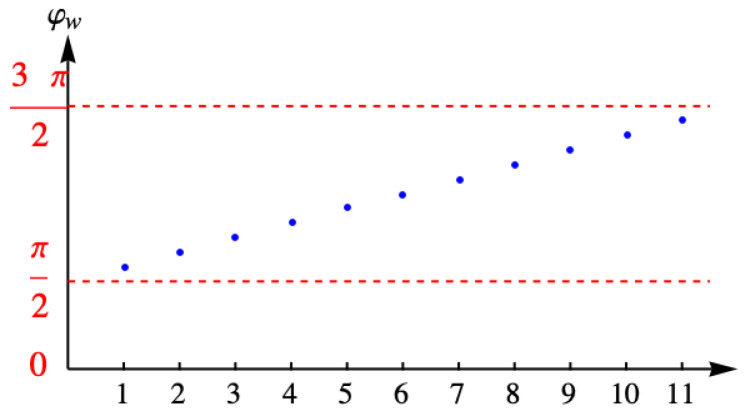
Discrete values of the angle $\varphi_{\omega }$ for the example with eleven columns.

Taking logarithms of both sides gives

(73) $$i\left(n_{c}+1\right)\varphi =-i\left(n_{c}+1\right)\left(\pi -\varphi \right)+2\pi i\omega ,$$

where *ω* is a winding number that gives the number of times that the point φ+2πω circles the singularity that terminates the branch cut of the logarithm of $e^{i(n_{c}+1)\varphi }$. As a result, the angle φ is quantized, that is, it acquires only discrete values given by

(74) $$2\left(n_{c}+1\right)\varphi =\left(n_{c}+1\right)\pi +2\pi \omega ,$$

so that φ only takes the values

(75) $$\varphi =\frac{\pi }{2}+\frac{\omega }{n_{c}+1}\pi ,$$

that is, the discrete values $\varphi =\frac{\pi }{2}+\frac{\pi }{n_{c}+1},\;\frac{\pi }{2}+\frac{2\pi }{n_{c}+1},\;\cdots ,\;\frac{\pi }{2}+\frac{n_{c}\pi }{n_{c}+1}$. The values φ=0 and φ=π are the boundary points where the solution vanishes that were added by padding the ends of the list. The components of the eigenvector $v_{k}^{\omega }$ are

(76) $$v_{k}^{\omega }=A\left[e^{ik\varphi_{\omega }}-{\left(-1\right)}^{k}e^{-ik\varphi_{\omega }}\right],$$

with *A* the normalization constant determined from $\sum_{k=1}^{n_{c}-1}{\left|v_{k}^{\omega }\right|}^{2}=1$. Figure 11 shows the discrete values of the angle $\varphi_{\omega }$ for the example with eleven columns.

The previous derivation of the eigenvalue λ is unchanged. Insert one of the terms of the generic solution $v_{k}=e^{\pm ik\varphi }$—either one will do—into the difference equation

(77) $$v_{k+1}-v_{k-1}=\frac{\lambda }{x}v_{k}.$$

Substituting $v_{k}=e^{ik\varphi }$ yields

(78) $$\frac{\lambda }{x}=e^{i\varphi }-e^{-i\varphi }=2i\sin\varphi ,$$

and as long as the eigenvalue λ satisfies this equation, $v_{k}=e^{\pm ik\varphi }$ is a solution to the difference equation. When we include the boundary-condition requirements, the angle φ is restricted to the discrete values $\varphi_{\omega }$, and the eigenvalues of *X* are

(79) $$\lambda_{\omega }=2i\sin\varphi_{\omega }.$$

**Figure 12:**
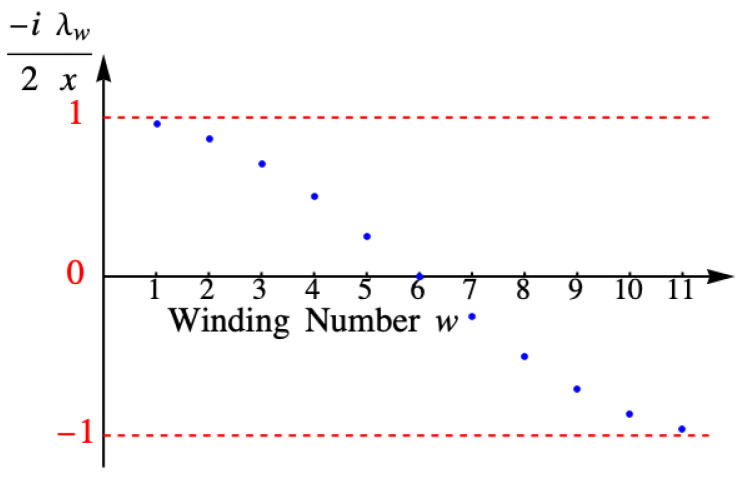
Eigenvalue $\lambda_{\omega }$ for the example with eleven columns.

Figure 12 shows the values of the eigenvalue $\lambda_{\omega }$ for the example with eleven columns.

The expression for the eigenfunction,

(80) $$v_{k}^{\omega }=A\left[e^{ik\varphi_{\omega }}-{\left(-1\right)}^{k}e^{-ik\varphi_{\omega }}\right],$$

can be rewritten by inserting the explicit form of the angle $\varphi_{\omega }=\frac{\pi }{2}+\frac{\omega \pi }{n_{c}+1}$. This gives

(81) $$\frac{1}{A}v_{k}^{\omega }=e^{ik(\frac{\pi }{2}+\frac{\omega \pi }{n_{c}+1})}-e^{i\pi k}e^{-ik(\frac{\pi }{2}+\frac{\omega \pi }{n_{c}+1})}=e^{i\frac{\pi }{2}k}\left[e^{ik\frac{\omega \pi }{n_{c}+1}}-e^{-ik\frac{\omega \pi }{n_{c}+1}}\right]=\left(i^{k}\right)\left[2i\sin\left(\frac{\pi \omega k}{n_{c}+1}\right)\right],$$

giving

(82) $$v_{k}^{\omega }=2Ai^{k+1}\sin\left(\frac{\pi \omega k}{n_{c}+1}\right).$$

Let us now find the normalization constant using $\sum_{k=1}^{n_{c}-1}{\left|v_{k}^{\omega }\right|}^{2}=1$. The components of the eigenvector are $v_{k}^{\omega }=A\left[e^{ik\varphi_{\omega }}-{\left(-1\right)}^{k}e^{-ik\varphi_{\omega }}\right]$ with $\varphi_{\omega }=\left(\frac{\pi }{2}+\frac{\omega \pi }{n_{c}+1}\right)$. Substituting the expression for $\varphi_{\omega }$ gives

(83) $$\begin{array}{ccc} {\left|v_{k}^{\omega }\right|}^{2} & = & {\left|A\right|}^{2}\left[e^{ik\varphi_{\omega }}-{\left(-1\right)}^{k}e^{-ik\varphi_{\omega }}\right]\left[e^{ik\varphi_{\omega }}-{\left(-1\right)}^{k}e^{-ik\varphi_{\omega }}\right]\\ & = & {\left|A\right|}^{2}\left[1-{\left(-1\right)}^{k}e^{2ik\varphi_{\omega }}-{\left(-1\right)}^{k}e^{-2ik\varphi_{\omega }}+{\left(-1\right)}^{2k}\right]. \end{array}$$

We then have

(84) $${\left|v_{k}^{\omega }\right|}^{2}={\left|A\right|}^{2}\left[1+{\left(-1\right)}^{2k}-2{\left(-1\right)}^{k}\cos\left(2k\varphi_{\omega }\right)\right].$$

Now, ${\left(-1\right)}^{2k}=1$ because 2k is always even, giving

(85) $${\left|v_{k}^{\omega }\right|}^{2}={\left|A\right|}^{2}\left[2-2{\left(-1\right)}^{2k}\cos\left(2k\varphi_{\omega }\right)\right],$$

where $i^{k}{\left(-i\right)}^{k}=1$. The normalization constant is then given by the finite sum

(86) $$\sum_{k=1}^{n_{c}}{\left|v_{k}^{\omega }\right|}^{2}=\sum_{k=1}^{n_{c}}{\left|A\right|}^{2}\left[2-2{\left(-1\right)}^{2k}\cos\left(2k\varphi_{\omega }\right)\right]=1,$$

which gives

(87) $$\frac{1}{2{\left|A\right|}^{2}}=\sum_{k=1}^{n_{c}}\left[1-{\left(-1\right)}^{k}\cos\left(2k\varphi_{\omega }\right)\right].$$

Then, since a shift in phase of kπ gives another factor of ${\left(-1\right)}^{k}$,

(88) $$\sum_{k=1}^{n_{c}}{\left(-1\right)}^{k}\cos\left(2k\varphi_{\omega }\right)=\sum_{k=1}^{n_{c}}{\left(-1\right)}^{2k}\cos\left[2k\left(\frac{\pi }{2}+\frac{\omega \pi }{n_{c}+1}\right)\right]=\sum_{k=1}^{n_{c}}\cos\left(\frac{2\pi k\omega }{n_{c}+1}\right).$$

The value of the sum over cosines is −1, and the normalization factor is

(89) $$\left|A\right|=\frac{1}{\sqrt{2(n_{c}+1)}}.$$

**Figure 13:**
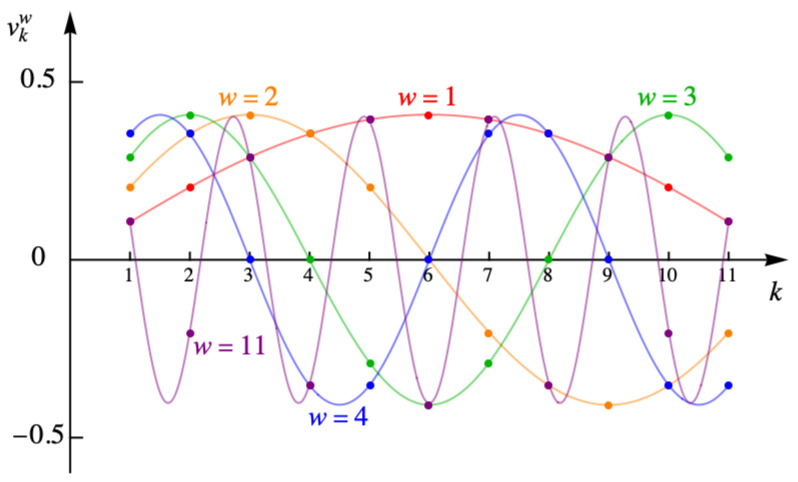
First four and the last eigenvectors $v_{k}^{\omega }$ for the example with eleven columns.

Taking A to be real and positive, the normalized eigenvectors are

(90) $$v_{k}^{\omega }=\frac{1}{\sqrt{2(n_{c}+1)}}\left[e^{ik\varphi_{\omega }}-{\left(-1\right)}^{k}e^{-ik\varphi_{\omega }}\right]=\sqrt{\frac{2}{n_{c}+1}}i^{k+1}\sin\left(\frac{\pi k\omega }{n_{c}+1}\right),$$

with $\varphi =\frac{\pi }{2}+\frac{\omega \pi }{n_{c}+1}$ and eigenvalue $\lambda_{\omega }=2ix\sin\varphi_{\omega }$. The subscript *k* distinguishes the various components of a given eigenvector $v^{\omega }$. The values φ=0 and π are the boundary points where $v_{k}^{\omega }$ vanishes. In Figure 13, are the first four eigenvectors for $n_{c}=11$ and various winding numbers *ω*. The points show the values, while the lines are merely to guide the eye.

The previous example of a lattice of sites that has three rows and four columns of sites was shown in Figure 6. This lattice has 3×4=12 sites, so the dimensions of the antisymmetric matrix *M* that gives the square of the partition function through

(91) $$Z^{2}={\left(\mathrm{Pf}\left[M\right]\right)}^{2}=\det\left[M\right]$$

is 12×12, that is, $N_{\mathrm{sites}}\times N_{\mathrm{sites}}$. This matrix *M* provides an example of the block-matrix form of the antisymmetric matrix *M* having the Pfaffian array as its upper right triangle, and is given in terms of the dimer activities *x* and *y* by the matrix shown in Figure 10.

This particular matrix has 12 rows and 12 columns, and consists of 3×3=9 blocks called *X*, *Y*, and zero. The matrix is divided into blocks by the red lines in Figure 10, and, in general, such a matrix has the form

(92) $$M=\left(\begin{array}{cccc} X & Y & & \\ -Y & X & \ddots & \\ & \ddots & \ddots & Y\\ & & -Y & X \end{array}\right).$$

For clarity, the zeros are not shown. This is a tridiagonal block matrix, and this form always arises when using serpentine numbering of the lattice sites. The size of each block is the number of sites along a single row of the lattice, as is most easily seen by examining the blocks *Y*. The number of blocks is the square of the number of rows in the lattice of sites.

Earlier, we described the diagonalization procedure for a matrix with the form of the block *X*. That same procedure can be used to find eigenvectors and eigenvalues of the block form of *M*. One can manipulate the blocks just like matrix elements, because block matrices can be multiplied block by block. The notation, though, can be confusing if the matrix, which is *M*, is denoted by *M* both when it is written as matrix elements and when it is written in blocks. Consequently, let us call the block form *B*, writing

(93) $$B=\left(\begin{array}{cccc} X & Y & & \\ -Y & X & \ddots & \\ & \ddots & \ddots & Y\\ & & -Y & X \end{array}\right)$$

when it is in block form, and

(94) $$M=\left(\begin{array}{cccc} 0 & x & & \\ -x & 0 & \ddots & \\ & \ddots & \ddots & x\\ & & -x & 0 \end{array}\right)$$

when it is written in terms of its matrix elements $m_{jk}=x,y,0$.

We will need here our earlier observation that, in general, when the lattice of sites has $n_{r}$ rows and $n_{c}$ columns, the dimension of each individual block is $n_{c}\times n_{c}$. The matrix of blocks, on the other hand, has the dimension $n_{r}\times n_{r}$. The total number of matrix elements is then $n_{r}^{2}n_{c}^{2}$, which is the square of the number of sites $N_{\mathrm{sites}}=n_{r}n_{c}$.

The eigenvectors of the block matrix are the vectors *u* with components $u_{j}$ that satisfy

(95) Bu=Λu.

The components $u_{j}$ will actually be vectors, so that a given $u_{j}$ may have components $v_{k}$ as in the eigenvectors of the block *X* given in Equation (90). That, however, does not concern us here. Eventually, though, the transformed matrix *M* will be cruciform.

Writing the eigenvector in terms of its components, the eigenvalue equation is

(96) $$\begin{array}{ccc} Bu & = & \left(\begin{array}{ccccc} X & Y & & & \\ -Y & X & & \ddots & \\ & & \ddots & & \\ & \ddots & & \ddots & Y\\ & & & -Y & X \end{array}\right)\left(\begin{array}{c} u_{1}\\ \vdots \\ u_{l}\\ \vdots \\ u_{n_{r}} \end{array}\right)=\left(\begin{array}{c} Xu_{1}+Yu_{2}+\cdots \\ \vdots \\ -Yu_{l-1}+Xu_{l}+Yu_{l+1}+\cdots \\ \vdots \\ -Yu_{n_{r}-1}+Xu_{n_{r}}++\cdots \end{array}\right)\\ & = & \lambda \left(\begin{array}{c} u_{1}\\ \vdots \\ u_{l}\\ \vdots \\ u_{n_{r}} \end{array}\right)=\lambda u. \end{array}$$

The eigenvectors can be found from the rows, which are given by

(97) $$-Yu_{l-1}+Xu_{l}+Yu_{l+1}=\Lambda u,$$

with boundary conditions

(98) $$u_{0}=u_{n_{r}+1}=0,$$

just as in the example of the diagonalization of the *X* matrix earlier in this section. Since the block matrix *B* is an $n_{r}\times n_{r}$ array of blocks, the eigenvector has $n_{r}$ components.

This difference equation has solution, once again, of the form $e^{il\theta }$ or, in order to keep the angle θ positive, a linear combination consisting of $e^{il\theta }$ and $e^{-il\theta }$. We write these eigenvectors as

(99) $$u_{l}=Ae^{il\theta }+B{\left(-1\right)}^{l}e^{-il\theta },$$

and apply the l=0 boundary condition to obtain

(100) $$u_{0}=A+B,$$

and so B=−A, and then

(101) $$u_{l}=A\left[e^{il\theta }-{\left(-1\right)}^{l}e^{-il\theta }\right]=0,$$

Either of the two terms appearing here could be used to find the eigenvalue Λ by inserting it into the difference equation. We choose $e^{il\theta }$, and have

(102) $$-Ye^{i(l-1)\theta }+Xe^{il\theta }+Ye^{i(l+1)\theta }=\Lambda e^{il\theta },$$

which, after canceling $e^{il\theta }$, gives

(103) $$\Lambda =-Ye^{-i\theta }+X+Ye^{i\theta },$$

so that

(104) Λ=X+2iYsinθ

is an $n_{c}\times n_{c}$ matrix that represents the “eigenvalue” of a block, irrespective of whether or not *X* and *Y* are diagonal or even simultaneously diagonalizable.

The second boundary condition $u_{m+1}=0$ quantizes the angle θ, just as it quantized the angle φ for the matrix *X*. It gives

(105) $$u_{n_{r}+1}=A\left[e^{i(n_{r}+1)\theta }-{\left(-1\right)}^{n_{r}+1}e^{-i(n_{r}+1)\theta }\right]=0,$$

so that

(106) $$e^{i(n_{r}+1)\theta }={\left(-1\right)}^{n_{r}+1}e^{-i(n_{r}+1)\theta }=e^{i\pi (n_{r}+1)}e^{-i(n_{r}+1)\theta }.$$

Taking the logarithm of both sides shows that

(107) $$\left(n_{r}+1\right)\theta =\left(n_{r}+1\right)\pi +2\pi W,$$

where *W* is another winding number arising from crossing the branch cut in the logarithm. This is what provides the complete set of eigenvectors, characterized by the various values that the angle $\theta =\theta_{W}$ can take on as *W* assumes the values $W=1,\;2,\;\cdots n_{r}$. Then,

(108) $$\theta_{W}=\frac{\pi }{2}+\frac{\pi W}{n_{r}+1}$$

are the various values of $\theta_{W}$. The values θ=0 and θ=π are the boundary points where the solution vanishes that were added by padding the ends of the list. The components of the eigenvector $u_{l}^{W}$ are

(109) $$u_{l}^{W}=A\left[e^{ik\theta_{W}}-{\left(-1\right)}^{l}e^{-ik\theta_{W}}\right],$$

with *A* the normalization constant determined from $\sum_{k=1}^{n_{c}-1}{\left|u_{k}^{W}\right|}^{2}=1$.

The expression for the eigenfunction,

(110) $$u_{l}^{W}=A\left[e^{il\theta_{W}}-{\left(-1\right)}^{l}e^{-il\theta_{W}}\right]=0,$$

can be rewritten by inserting the explicit form of the angle $\theta_{W}=\frac{\pi }{2}+\frac{W\pi }{n_{r}+1}$. This produces

(111) $$\frac{1}{A}u_{l}^{W}=e^{il(\frac{\pi }{2}+\frac{W\pi }{n_{r}+1})}-e^{i\pi l}e^{-il(\frac{\pi }{2}+\frac{W\pi }{n_{r}+1})}=e^{i\frac{\pi }{2}l}\left[e^{il\frac{W\pi }{n_{r}+1}}-e^{-il\frac{W\pi }{n_{r}+1}}\right]={\left(i\right)}^{l}\left[2i\sin\left(\frac{\pi Wl}{n_{r}+1}\right)\right],$$

giving

(112) $$u_{l}^{W}=2A{\left(i\right)}^{l+1}\sin\left(\frac{\pi lW}{n_{r}+1}\right).$$

The normalization constant *A* is found earlier in this section in the diagonalization of the *X* matrix by requiring that $\sum_{l=1}^{n_{r}}{\left|u_{l}^{W}\right|}^{2}=1$. This gives

(113) $$A=\frac{1}{\sqrt{2(n_{r}+1)}},$$

and so the components of the normalized eigenvector are

(114) $$u_{l}^{W}=\sqrt{\frac{2}{n_{r}+1}}i^{l+1}\sin\left(\frac{\pi lW}{n_{r}+1}\right),$$

and the associated eigenvalue is

(115) $$\Lambda_{W}=X+2iY\sin\theta_{W}.$$

These are the block eigenvalues $\Lambda_{W}$ of the block matrix *B*. Writing these down the diagonal gives *B* in diagonal form,

(116) $$B=\left(\begin{array}{ccccc} X+2iY\sin(\theta_{1}) & & & & \\ & X+2iY\sin(\theta_{2}) & & & \\ & & X+2iY\sin(\theta_{3}) & & \\ & & & \ddots & \\ & & & & X+2iY\sin(\theta_{n_{r}}) \end{array}\right).$$

Each of these eigenvalues is actually an $n_{c}\times n_{c}$ matrix forming a block of the now-diagonal block matrix *B*, and if written out in full represents the original $N_{\mathrm{sites}}\times N_{\mathrm{sites}}$ matrix *M*, although that matrix is not yet cruciform. We now set out to make it so.

The block *X* is not yet in diagonal form, although we know its eigenvalues, which are $\lambda_{\omega }=2ix\sin\varphi_{\omega }$ with $\omega =1,2,\cdots n_{c}$. Thus, when diagonalized, we have

(117) $$X=\left(\begin{array}{ccccc} 2ix\sin(\varphi_{1}) & & & & \\ & 2ix\sin(\varphi_{2}) & & & \\ & & 2ix\sin(\varphi_{3}) & & \\ & & & \ddots & \\ & & & & 2ix\sin(\varphi_{n_{c}}) \end{array}\right).$$

The question is, what happens to *Y* when we transform both *X* and *Y* using the unitary transformation *U* that diagonalizes *X*? The short answer is that the vertical dimer activity *y* shows up along the antidiagonal, which is the “diagonal” that runs from the lower left to upper right corners of a matrix. This produces the cruciform matrix

(118) $$\Lambda_{W}=\left(\begin{array}{cccccc} \lambda_{1} & & & & & {\left(-1\right)}^{n_{c}-1}\xi \\ & \lambda_{2} & & & {\left(-1\right)}^{n_{c}-2}\xi & \\ & & \ddots & ⋰ & & \\ & & ⋰ & \ddots & & \\ & {\left(-1\right)}^{1}\xi & & & \lambda_{n_{c}-1} & \\ {\left(-1\right)}^{0}\xi & & & & & \lambda_{n_{c}} \end{array}\right),$$

where $\xi =2i^{n_{c}}y\sin\theta_{W}$. We then have a block matrix consisting of these cruciform blocks, and its determinant gives the square of the partition function.

This cruciform shape results from the transformation *U* that diagonalizes *X*, which is constructed from the normalized eigenvectors $v^{\omega }$ of *X* with the components of each vector $v^{\omega }$ forming the columns of *U*, giving

(119) $$U=\left(\begin{array}{ccccc} v_{1}^{1} & \cdots & v_{1}^{\omega } & \cdots & v_{1}^{n_{c}}\\ \vdots & \ddots & & & \\ v_{k}^{1} & & v_{k=\omega }^{\omega } & & \\ \vdots & & & \ddots & \\ v_{n_{c}}^{1} & & & & v_{n_{c}}^{n_{c}} \end{array}\right).$$

The block *Y*, written explicitly in terms of the activity, has entries *y* along the antidiagonal and can be written as *y* times the matrix with entries of 1 on the antidiagonal. To see what effect such a matrix has on another matrix, a two-dimensional example should be sufficient. We have

(120) $$\begin{array}{ccc} \frac{1}{y}U^{†}YU & = & \left(\begin{array}{cc} u_{11}^{*} & u_{21}^{*}\\ u_{12}^{*} & u_{22}^{*} \end{array}\right)\left(\begin{array}{cc} 0 & 1\\ 1 & 0 \end{array}\right)\left(\begin{array}{cc} u_{11} & u_{12}\\ u_{21} & u_{22} \end{array}\right)=\left(\begin{array}{cc} u_{11}^{*} & u_{21}^{*}\\ u_{12}^{*} & u_{22}^{*} \end{array}\right)\left(\begin{array}{cc} u_{21} & u_{22}\\ u_{11} & u_{12} \end{array}\right)\\ & = & \left(\begin{array}{cc} u_{11}^{*}u_{21}+u_{21}^{*}u_{11} & u_{11}^{*}u_{22}+u_{21}^{*}u_{12}\\ u_{12}^{*}u_{21}+u_{22}^{*}u_{11} & u_{12}^{*}u_{22}+u_{22}^{*}u_{12} \end{array}\right). \end{array}$$

It seems that the effect of a matrix with unity along the antidiagonal (the anti-identity?) is to reverse the rows of the matrix it multiplies. This result implies that the matrix element $t_{12}$ of $\frac{1}{y}U^{†}YU$ is

(121) $$t_{12}=u_{11}^{*}u_{n_{c}2}+u_{21}^{*}u_{(n_{c}-1)2}+u_{31}^{*}u_{(n_{c}-2)2}+\cdots +u_{k1}^{*}u_{(n_{c}-k+1)2}+\cdots +u_{n_{c}1}^{*}u_{(n_{c}-n_{c}+1)2}.$$

The general matrix element then consists of the product of the component $u_{k\omega }^{*}$ and $u_{k^{\prime }\omega^{\prime }}$ with $k^{\prime }=n_{c}-k+1$, or in terms of the components of the eigenvector, $v_{k}^{\omega *}$ and $v_{k^{\prime }=n_{c}-k+1}^{\omega^{\prime }}$. The matrix elements of $\frac{1}{y}U^{†}YU$ are

(122) $$t_{\omega ,\omega^{\prime }}=\sum_{k=1}^{n_{c}}v_{k}^{\omega *}v_{n_{c}-k+1}^{n_{c}-\omega }=i^{n_{c}-1}{\left(-1\right)}^{n_{c}-\omega }\delta_{\omega^{\prime },n_{c}+1-\omega },$$

which is derived in detail in Appendix D.

These are the terms that appear on the antidiagonal of *Y* after transforming to the representation in which *X* is diagonal. All matrix elements off the antidiagonal of *Y* are zero. This $n_{c}\times n_{c}$ matrix *Y* can finally be written as

(123) $$Y=y\left(\begin{array}{cccc} & & & i^{n_{c}-1}{\left(-1\right)}^{n_{c}-1}\\ & & \cdots & \\ & i^{n_{c}-1}{\left(-1\right)}^{n_{c}-\left(n_{c}-1\right)} & & \\ i^{n_{c}-1}{\left(-1\right)}^{n_{c}-n_{c}} & & \end{array}\right).$$

The transformed matrix $\Lambda_{W}=X+2i\sin\left(\theta_{W}\right)Y$ becomes

(124) $$\Lambda_{W}=\left(\begin{array}{cccccc} 2ix\sin(\varphi_{1}) & & & & & {\left(-1\right)}^{n_{c}-1}\xi )\\ & 2ix\sin(\varphi_{2}) & & & {\left(-1\right)}^{n_{c}-2}\xi & \\ & & \ddots & ⋰ & & \\ & & ⋰ & \ddots & & \\ & {\left(-1\right)}^{1}\xi & & & 2ix\sin(\varphi_{n_{c}-1}) & \\ {\left(-1\right)}^{0}\xi & & & & & 2ix\sin(\varphi_{n_{c}}) \end{array}\right).$$

This matrix $\Lambda_{W}$ is a block of the matrix *B*, which, because it has the form of a cross, is called a cruciform matrix.

The partition function is given by the Pfaffian of this matrix *B*, and since the square of a Pfaffian is a determinant, it is more convenient to evaluate the square of the partition function, which is the determinant of *B*. The block eigenvalues $\Lambda_{W}$ form the diagonal elements of *B*, while all other blocks are zero. The determinant of a diagonalized block matrix like this is the product of the determinants of the individual blocks, and the square of the partition function is the determinant.

(125) $$Z^{2}=\det\left[B\right]=\prod_{W=1}^{n_{r}}\det\Lambda_{W}.$$

The determinant of a cruciform matrix is easily determined by expanding by the first row of the matrix and then in each minor expanding by the last row. Then each new minor is expanded by the first row and each of the resulting minors by their bottom rows. This repetition creates $\left\lfloor \frac{1}{2}\left(n+1\right)\right\rfloor$ factors of the type $\left(d_{11}d_{nn}-d_{1n}d_{n1}\right)$. The brackets ⌊x⌋ indicate the floor function, which gives the highest integer lower than *x*. The square of the partition function is evaluated in Appendix E, with the result

(126) $$Z^{2}={\left(-1\right)}^{n_{r}\left\lfloor \frac{n_{c}}{2}\right\rfloor }\prod_{\omega =1}^{n_{c}}\prod_{W=1}^{n_{r}}\left[2x\cos\left(\frac{\pi \omega }{n+1}\right)+2iy\cos\left(\frac{\pi W}{n_{r}+1}\right)\right].$$

The square root of this, the partition function itself, is also evaluated in Appendix E and is given by

(127) $$Z=2^{\frac{n_{c}}{2}\left\lfloor \frac{n_{r}}{2}\right\rfloor }\prod_{\omega =1}^{\frac{n_{c}}{2}}\prod_{W=1}^{\left\lfloor \frac{n_{r}}{2}\right\rfloor }\left[x^{2}\left(1+\cos\left(\frac{2\pi \omega }{n_{c}+1}\right)\right)+y^{2}\left(1+\cos\left(\frac{2\pi W}{n_{r}+1}\right)\right)\right]\times \left\{\begin{array}{cc} 1 & n_{r}\;\mathrm{even}\\ x^{\frac{n_{c}}{2}} & n_{r}\;\mathrm{odd} \end{array}\right..$$

## 8. Dimer Partition Function in the Large Lattice Limit

We can calculate the partition function explicitly if we take the limit as the lattice becomes large, $n_{r}\to \infty$ and $n_{c}\to \infty$. It is better to find this limit of the logarithm of Z, so that products become sums and we have

(128) $$\begin{array}{ccc} \ln Z & = & \ln\left(2^{\frac{n_{c}}{2}\left\lfloor \frac{n_{r}}{2}\right\rfloor }\right)+\sum_{\omega =1}^{\frac{n_{c}}{2}}\sum_{W=1}^{\left\lfloor \frac{n_{r}}{2}\right\rfloor }\ln\left\{x^{2}\left[1+\cos\left(\frac{2\pi \omega }{n_{c}+1}\right)\right]+y^{2}\left[1+\cos\left(\frac{2\pi W}{n_{r}+1}\right)\right]\right\}+\\ & & +\left\{\begin{array}{cc} 0 & n_{r}\;\mathrm{even}\\ \frac{n_{c}}{2}\ln x & n_{r}\;\mathrm{odd} \end{array}\right.. \end{array}$$

In the infinite limit, $n_{r}$ and $n_{c}$ are much greater than unity, and so $n_{r}+1∼n_{r}$ and $n_{c}+1∼n_{c}$, while $\left\lfloor \frac{n_{r}}{2}/2\right\rfloor ∼\frac{n_{r}}{2}$. The basic rule for converting sums to integrals is

(129) $$\sum_{k=1}^{k_{\max}}f\left(x_{k}\right)\Delta x=\int_{a}^{b}f\left(x\right)dx,$$

with $\Delta x=\frac{b-a}{k_{\max}-1}$ and $x_{k}=a+\left(k-1\right)\Delta x$. Using this, the sum over *ω* becomes

(130) $$\sum_{\omega =1}^{\frac{n_{c}}{2}}\ln\left\{x^{2}\left[1+\cos\left(\frac{2\pi \omega }{n_{c}+1}\right)\right]+y^{2}\left[1+\cos\left(\frac{2\pi W}{n_{r}+1}\right)\right]\right\}∼\int_{a}^{b}f\left(x\right)dx,$$

Choosing the integration variable as $t=\frac{2\pi \omega }{n_{c}}$ is convenient, giving a factor $dt=\frac{dt}{d\omega }d\omega =\frac{2\pi }{n_{c}}d\omega$, and when $n_{c}$ is large, the lower limit ω=1 becomes a≈0. The upper limit is $b=\frac{2\pi }{n_{c}}\frac{n_{c}}{2}=\pi$, and so the integral takes the form

(131) $$\begin{array}{ccc} & & \sum_{\omega =1}^{\frac{n_{c}}{2}}\ln\left\{x^{2}\left[1+\cos\left(\frac{2\pi \omega }{n_{c}+1}\right)\right]+y^{2}\left[1+\cos\left(\frac{2\pi W}{n_{r}+1}\right)\right]\right\}\\ & & ∼\int_{0}^{\pi }\frac{n_{c}}{2\pi }dt\ln\left\{x^{2}\left[1+\cos t\right]+y^{2}\left[1+\cos\left(\frac{2\pi W}{n_{r}}\right)\right]\right\}. \end{array}$$

The analog for the sum over *W* is

(132) $$\begin{array}{ccc} & & \sum_{W=1}^{\frac{n_{r}}{2}}\ln\left\{x^{2}\left[1+\cos\left(\frac{2\pi \omega }{n_{c}+1}\right)\right]+y^{2}\left[1+\cos\left(\frac{2\pi W}{n_{r}+1}\right)\right]\right\}\\ & & ∼\int_{0}^{\pi }\frac{n_{r}}{2\pi }ds\ln\left\{x^{2}\left[1+\cos\left(\frac{2\pi \omega }{n_{c}}\right)\right]+y^{2}\left[1+\cos s\right]\right\}. \end{array}$$

The double sum is then

(133) $$\begin{array}{ccc} & & \sum_{\omega =1}^{\frac{n_{c}}{2}}\sum_{W=1}^{\frac{n_{r}}{2}}\ln\left\{x^{2}\left[1+\cos\left(\frac{2\pi \omega }{n_{c}+1}\right)\right]+y^{2}\left[1+\cos\left(\frac{2\pi W}{n_{r}+1}\right)\right]\right\}\\ & & ∼\frac{n_{c}n_{r}}{4\pi^{2}}\int_{0}^{\pi }ds\int_{0}^{\pi }dt\ln\left\{x^{2}\left[1+\cos t\right]+y^{2}\left[1+\cos s\right]\right\}. \end{array}$$

The integral is given by Equation (4.224.9) of reference [16] as

(134) $$\int_{0}^{\pi }ds\ln\left(a+b\cos s\right)=\pi \ln\left(\frac{a+\sqrt{a^{2}-b^{2}}}{2}\right)\;\mathrm{for}\;a\geq \left|b\right|>b.$$

Here, $a=x^{2}\left(1+\cos t\right)+y^{2}$ or $y^{2}\left(1+\cos s\right)$, so that the integrals can be evaluated, although we have no need to do so. The entropy is an extensive quantity, with its logarithm proportional to $N_{\mathrm{sites}}$ as the number of sites becomes large. The result diverges as the lattice becomes infinite, and the only meaningful expression is the “partition function per site”,

(135) $$\begin{array}{ccc} \ln Z_{\mathrm{site}} & \equiv & \lim_{N_{\mathrm{sites}}\to \infty }\frac{1}{N_{\mathrm{sites}}}\sum_{\omega =1}^{\frac{n_{c}}{2}}\sum_{W=1}^{\frac{n_{r}}{2}}\ln\left\{x^{2}\left[1+\cos\left(\frac{2\pi \omega }{n_{c}+1}\right)\right]+y^{2}\left[1+\cos\left(\frac{2\pi W}{n_{r}+1}\right)\right]\right\}. \end{array}$$

This result is given by Fisher and followed by a derivation of the entropy and an excellent discussion of the result and associated dimer densities per site $n_{x}$ and $n_{y}$ that will not be reiterated here [7].

## 9. Colored *y* Dimers and the Partition Function for Dimers and Monomers

**Figure 14:**
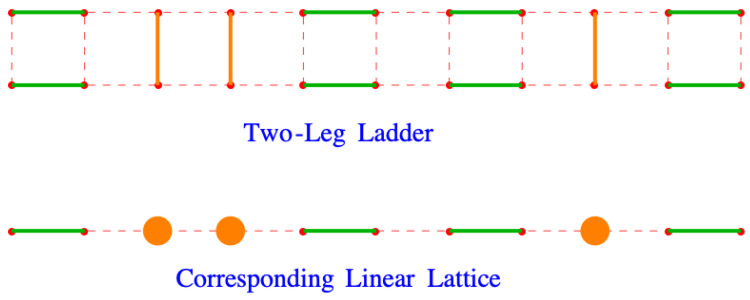
A two-leg ladder ((**top**) figure) viewed from above ((**bottom**) figure). The *y* dimers are shown in orange, with the *x* dimers shown in green. The bottom picture could be considered to be a linear lattice in which the orange *y* dimers appear to be monomers. The red dots and dashes outline the lattices.

The partition function in Equation (127) is applicable to any lattice formed from $n_{r}$ rows and $n_{c}$ columns of lattice points. Suppose that the lattice has the form of a two-leg ladder, with $n_{r}=2$ rows and $n_{c}$ columns and that it is completely filled with dimers, ν=1. A dimer arrangement on this lattice is shown in the top of Figure 14. The *y*-direction dimers are colored orange to distinguish them from the *x* dimers, which are colored green. Looking down on this ladder so that it is viewed edge-on, as in the bottom picture, it looks like a linear lattice filled with dimers and orange monomers.

What is the effect of randomly coloring the vertical dimers red and blue and assigning them corresponding activities $y_{r}$ and $y_{b}$, just like the red and blue balls discussed earlier? If we could find the partition function for this system, we could have a linear system of dimers, monomers, and vacant sites, even if this problem has not been solved analytically in two dimensions in Fisher’s work.

We can see how to do this by starting with the expression

(136) $$Z=\sum_{N_{x},\;N_{y}}g\left(N_{x},N_{y}\right)x^{N_{x}}y^{N_{y}},$$

because $g(N_{x},\;N_{y})$ is the number of states accessible to the system when there are precisely $N_{x}$ horizontal dimers and $N_{y}$ vertical dimers. If the vertical y-dimers come in two colors, $y_{r}$ and $y_{b}$, the number of distinct states increases, and does so by a factor of $\frac{N_{y}!}{N_{r}!N_{b}!}$, where $N_{r}$ is the number of red *y*-bonds and $N_{b}$ is the number of blue *y*-dimers, and $N_{r}+N_{b}=N_{y}$. The sum over $N_{y}$ gains the additional factor

(137) $$\sum_{N_{r}=0}^{N_{y}}\frac{N_{y}!}{N_{r}!N_{b}!}y_{r}^{N_{r}}y_{b}^{N_{b}}=\sum_{N_{r}=0}^{N_{y}}\frac{N_{y}!}{N_{r}!\left(N_{y}-N_{r}\right)!}y_{r}^{N_{r}}y_{b}^{N_{y}-N_{r}}={\left(y_{r}+y_{b}\right)}^{N_{y}},$$

and we have

(138) $$Z=\sum_{N_{x},\;N_{y}}g\left(N_{x},N_{y}\right)x^{N_{x}}\sum_{N_{r}=0}^{N_{y}}\frac{N_{y}!}{N_{r}!N_{b}!}y_{r}^{N_{r}}y_{b}^{N_{b}}=\sum_{N_{x},\;N_{y}}g\left(N_{x},N_{y}\right)x^{N_{x}}{\left(y_{r}+y_{b}\right)}^{N_{y}}.$$

The link fields $A^{\mu }$ are colorless and do not distinguish between $y_{r}$ and $y_{b}$, even though they throw away a large fraction of the random arrangements of the child’s toys. Thus, the rest of the calculation proceeds as before, the only change being the replacement $y\to y_{r}+y_{b}$, giving

(139) $$\begin{array}{ccc} Z & = & 2^{\frac{n_{c}}{2}\left\lfloor \frac{n_{r}}{2}\right\rfloor }\prod_{\omega =1}^{\frac{n_{c}}{2}}\prod_{W=1}^{\left\lfloor \frac{n_{r}}{2}\right\rfloor }\left[x^{2}\left(1+\cos\left(\frac{2\pi \omega }{n_{c}+1}\right)\right)+{\left(y_{r}+y_{b}\right)}^{2}\left(1+\cos\left(\frac{2\pi W}{n_{r}+1}\right)\right)\right]\\ & & \times \left\{\begin{array}{cc} 1 & n_{r}\;\mathrm{even}\\ x^{\frac{n_{c}}{2}} & n_{r}\;\mathrm{odd} \end{array}\right.. \end{array}$$

We now return to the two-leg ladder. Here, we want a very long ladder, $n_{c}\to \infty$, while for two legs, $n_{r}=2$. Evaluating the partition function for $n_{r}=2$ gives $\lfloor \frac{n_{r}}{2}\rfloor =1$, and we have the logarithm of the partition function given by

(140) $$\begin{array}{ccc} \ln Z & = & \ln\left(2^{\frac{n_{c}}{2}\left\lfloor \frac{n_{r}}{2}\right\rfloor }\right)+\sum_{\omega =1}^{\frac{n_{c}}{2}}\sum_{W=1}^{\left\lfloor \frac{n_{r}}{2}\right\rfloor }\ln\left\{x^{2}\left[1+\cos\left(\frac{2\pi \omega }{n_{c}+1}\right)\right]+y^{2}\left[1+\cos\left(\frac{2\pi W}{n_{r}+1}\right)\right]\right\}+\\ & & +\left\{\begin{array}{cc} 0 & n_{r}\;\mathrm{even}\\ \frac{n_{c}}{2}\ln x & n_{r}\;\mathrm{odd} \end{array}\right.\\ & = & \ln2^{\frac{n_{c}}{2}}+\sum_{\omega =1}^{\frac{n_{c}}{2}}\sum_{W=1}^{1}\ln\left\{x^{2}\left[1+\cos\left(\frac{2\pi \omega }{n_{c}+1}\right)\right]+y^{2}\left[1+\cos\left(\frac{2\pi W}{2+1}\right)\right]\right\}\\ & = & \frac{n_{c}}{2}\ln2+\sum_{\omega =1}^{\frac{n_{c}}{2}}\ln\left\{x^{2}\left[1+\cos\left(\frac{2\pi \omega }{n_{c}+1}\right)\right]+y^{2}\left[1+\cos\left(\frac{2\pi }{3}\right)\right]\right\}. \end{array}$$

The angle $\frac{2\pi }{3}$ is $120^{\circ }$, and so the cosine is $-\frac{1}{2}$, giving

(141) $$\ln Z=\frac{n_{c}}{2}\ln2+\sum_{\omega =1}^{\frac{n_{c}}{2}}\ln\left\{x^{2}\left[1+\cos\left(\frac{2\pi \omega }{n_{c}+1}\right)\right]+\frac{1}{2}y^{2}\right\}.$$

Combining the first term with the sum brings a factor of two inside the log in the sum, giving

(142) $$\ln Z=\sum_{\omega =1}^{\frac{n_{c}}{2}}\ln\left\{2x^{2}\left[1+\cos\left(\frac{2\pi \omega }{n_{c}+1}\right)\right]+y^{2}\right\}.$$

**Figure 15:**
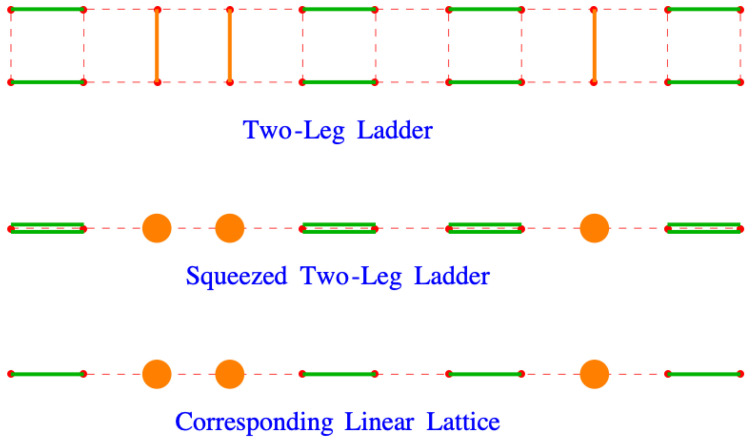
Imagine squeezing the rungs of the ladder together, producing the orange disks from the original y-oriented dimers.

This is the correct result for the two-leg ladder. It is not, however, correct for the linear chain. To see why, imagine squeezing the rungs of the ladder together, producing the orange disks from the original *y*-oriented dimers, as shown in Figure 15. This results in a double dimer, one from each leg of the ladder, on pairs of sites that should only a single dimer. In the logarithm of the partition function, the origin of the double dimers is the $x^{2}$ term, which because we have logarithms, gives $\ln x^{2}=\ln x+\ln x$. The correct result for the linear chain, $n_{r}=1$, has $x^{2}$ replaced by *x*,

(143) $$\ln Z=\sum_{\omega =1}^{\frac{n_{c}}{2}}\ln\left\{2x\left[1+\cos\left(\frac{2\pi \omega }{n_{c}+1}\right)\right]+y^{2}\right\}\;\mathrm{when}\;n_{r}=1.$$

Fisher implies that he confirmed this result by including vacancies in their original formulation and evaluating the Pfaffian, and states that this result is consistent with other ways of determining configurations of a monomer–dimer linear chain.

## 10. The Linear Chain

This gives the partition function for an infinitely long linear chain by letting $n_{c}\to \infty$ and using the integral representation introduced above. This gives

(144) $$\begin{array}{ccc} \ln Z_{\mathrm{site}} & = & \lim_{n_{c}\to \infty }\frac{1}{n_{c}}\sum_{\omega =1}^{\frac{n_{c}}{2}}\ln\left\{2x\left[1+\cos\left(\frac{2\pi \omega }{n_{c}+1}\right)\right]+y^{2}\right\}\\ & = & \int_{0}^{\pi }\frac{dt}{2\pi }\ln\left[2x\left(1+\cos t\right)+y^{2}\right]. \end{array}$$

Evaluating the integral gives $\pi \ln\left(\frac{a+\sqrt{a^{2}+b^{2}}}{2}\right)$ where $a=2x+y^{2}$ and b=2x. In the infinite limit of a very long chain,

(145) $$\ln Z_{\mathrm{site}}=\frac{1}{2}\ln\left(\frac{2x+y^{2}+\sqrt{{\left(2x+y^{2}\right)}^{2}-{\left(2x\right)}^{2}}}{2}\right).$$

Under the square root, we have $4x^{2}+4xy^{2}+y^{4}-4x^{2}=y^{2}\left(4x+y^{2}\right)$, giving

(146) $$\begin{array}{ccc} \ln Z_{\mathrm{site}} & = & \frac{1}{2}\ln\left(\frac{4x+2y\sqrt{4x+y^{2}}+2y^{2}}{4}\right)=\frac{1}{2}\ln\left(\frac{\left(4x+y^{2}\right)+2y\sqrt{4x+y^{2}}+y^{2}}{4}\right)\\ & = & \frac{1}{2}\ln\left({\left[\frac{\sqrt{4x+y^{2}}+y}{2}\right]}^{2}\right). \end{array}$$

Then the log of the partition function per site is

(147) $$\ln Z_{\mathrm{site}}=\ln\left(\frac{\sqrt{4x+y^{2}}+y}{2}\right)\;n_{r}=1,\;n_{c}\to \infty$$

for a long monomer–dimer chain. This allows systems involving the attachment of monomers and dimers to a long polymeric chain, such as charged dimers and point-like ions binding electrostatically to a DNA strand.

For colored vertical dimers, we replace the activity y by $y_{b}+y_{r}$, with $y_{r}$ the activity of red *y*-dimers and *y* the activity of blue y-dimers. Making this change in the partition function of the one-dimensional very long chain, we have

(148) $$\ln Z_{\mathrm{site}}=\ln\left(\frac{\sqrt{4x+{\left(y_{r}+y_{b}\right)}^{2}}+y_{r}+y_{b}}{2}\right)\;n_{r}=1,\;n_{c}\to \infty .$$

We can now identify the red *y*-dimers with monomers with activity $z_{⊥}=e^{-\beta (\varepsilon_{⊥}-\mu_{⊥})}$ and the blue *y*-dimers with vacancies with activity v=1, because vacancies have neither energy nor chemical potential, and so $v=e^{-\beta (\varepsilon_{v}-\mu_{v})}=e^{0}=1$. We will call the activity of the *x* dimers $x_{\|}=e^{-\beta (\varepsilon_{\|}-\mu_{\|})}$. Then the log of the partition function per site that includes *y* dimers and vacancies is

(149) $$\ln Z_{\mathrm{site}}=\ln\left(\frac{1}{2}\left[z_{⊥}+v+\sqrt{{\left(z_{⊥}+v\right)}^{2}+4x_{\|}}\right]\right),\;n_{r}=1,n_{c}\to \infty .$$

In general, the entropy per site is given by the expression

(150) $$S_{\mathrm{site}}=k_{B}\ln Z_{\mathrm{site}}-\frac{k_{B}\beta }{Z_{\mathrm{site}}}\frac{\partial Z_{\mathrm{site}}}{\partial \beta }.$$

Therefore, the entropy for the dimer model becomes

(151) $$S_{\mathrm{site}}=k_{B}\ln Z_{\mathrm{site}}-\frac{1}{2}\frac{k_{B}\beta }{Z_{\mathrm{site}}}\frac{\partial }{\partial \beta }\left(z_{⊥}+v+\sqrt{{\left(z_{⊥}+v\right)}^{2}+4x_{\|}}\right),$$

where twice the derivative of the partition function is

(152) $$\begin{array}{ccc} 2\frac{\partial }{\partial \beta }Z_{\mathrm{site}} & = & \frac{\partial }{\partial \beta }\left(\left(z_{⊥}+v\right)+\sqrt{{\left(z_{⊥}+v\right)}^{2}+4x_{\|}}\right)=\frac{\partial z_{⊥}}{\partial \beta }+\frac{\partial v}{\partial \beta }\\ & & +\frac{1}{2}{\left[{\left(z_{⊥}+v\right)}^{2}+4x_{\|}\right]}^{-\frac{1}{2}}\frac{\partial }{\partial \beta }\left[{\left(z_{⊥}+v\right)}^{2}+4x_{\|}\right]. \end{array}$$

The derivative of the activity $a=e^{-\beta (\varepsilon_{a}-\mu_{a})}$ with respect to β is

(153) $$\frac{\partial }{\partial \beta }a=-\left(\varepsilon_{a}-\mu_{a}\right)a=\frac{1}{\beta }a\ln a.$$

Therefore, the derivative of the partition function per site is

(154) $$\begin{array}{ccc} 2\frac{\partial }{\partial \beta }Z_{\mathrm{site}} & = & \frac{1}{\beta }z_{⊥}\ln z_{⊥}+0\\ & & +\frac{1}{2}{\left[{\left(z_{⊥}+v\right)}^{2}+4x_{\|}\right]}^{-\frac{1}{2}}\left[2\left(z_{⊥}+v\right)\frac{1}{\beta }z_{⊥}\ln z_{⊥}+\frac{4}{\beta }x_{\|}\ln x_{\|}\right]. \end{array}$$

This means that the entropy is given by

(155) $$\begin{array}{ccc} S_{\mathrm{site}} & = & k_{B}\ln Z_{\mathrm{site}}-\frac{1}{2}\frac{k_{B}\beta }{Z_{\mathrm{site}}}\left\{\frac{1}{\beta }z_{⊥}\ln z_{⊥}+\frac{1}{2}{\left[{\left(z_{⊥}+v\right)}^{2}+4x_{\|}\right]}^{-\frac{1}{2}}\times \right.\\ & & \left.\times \left[2\left(z_{⊥}+v\right)\frac{1}{\beta }z_{⊥}\ln z_{⊥}+\frac{4}{\beta }x_{\|}\ln x_{\|}\right]\right\}. \end{array}$$

Simplifying, we have

(156) $$\begin{array}{ccc} S_{\mathrm{site}} & = & k_{B}\ln Z_{\mathrm{site}}-\frac{k_{B}}{2Z_{\mathrm{site}}}\left\{z_{⊥}\ln z_{⊥}+{\left[{\left(z_{⊥}+v\right)}^{2}+4x_{\|}\right]}^{-\frac{1}{2}}\times \right.\\ & & \left.\times \left[\left(z_{⊥}+v\right)z_{⊥}\ln z_{⊥}+2x_{\|}\ln x_{\|}\right]\right\}. \end{array}$$

Similarly, the mean site occupation by the perpendicular dimers $z_{⊥}$ is

(157) $$n_{⊥}=\frac{1}{\beta Z_{\mathrm{site}}}\frac{\partial Z_{\mathrm{site}}}{\partial \mu_{⊥}}=\frac{1}{2\beta Z_{\mathrm{site}}}\frac{\partial }{\partial \mu_{⊥}}\left(z_{⊥}+v+\sqrt{{\left(z_{⊥}+v\right)}^{2}+4x_{\|}}\right).$$

Therefore, taking the derivative, we have

(158) $$n_{⊥}=\frac{1}{2\beta Z_{\mathrm{site}}}\left(\frac{\partial z_{⊥}}{\partial \mu_{⊥}}+0+\frac{1}{2}{\left[{\left(z_{⊥}+v\right)}^{2}+4x_{\|}\right]}^{-\frac{1}{2}}\frac{\partial }{\partial \mu_{⊥}}\left[{\left(z_{⊥}+v\right)}^{2}+4x_{\|}\right]\right).$$

The derivative of the activity $a=e^{-\beta (\varepsilon_{a}-\mu_{a})}$ with respect to $\mu_{a}$ is

(159) $$\frac{\partial }{\partial \mu_{a}}=\frac{\partial }{\partial \mu_{a}}e^{-\beta (\varepsilon_{a}-\mu_{a})}=\beta a,$$

and this is zero applied to the activity of a different type of particle. Then the mean site occupation by the perpendicular dimers $z_{⊥}$ is

(160) $$n_{⊥}=\frac{1}{2\beta Z_{\mathrm{site}}}\left(\beta z_{⊥}+\frac{1}{2}{\left[{\left(z_{⊥}+v\right)}^{2}+4x_{\|}\right]}^{-\frac{1}{2}}\left[2\left(z_{⊥}+v\right)\beta z_{⊥}\right]\right).$$

Simplifying, we have

(161) $$n_{⊥}=\frac{z_{⊥}}{2Z_{\mathrm{site}}}\left(1+\left(z_{⊥}+v\right){\left[{\left(z_{⊥}+v\right)}^{2}+4x_{\|}\right]}^{-\frac{1}{2}}\right).$$

Factoring out ${\left[{\left(z_{⊥}+v\right)}^{2}+4x_{\|}\right]}^{-\frac{1}{2}}$ from the parentheses, we have

(162) $$n_{⊥}=\frac{z_{⊥}}{2\sqrt{{\left(z_{⊥}+v\right)}^{2}+4x_{\|}}}\frac{1}{Z_{\mathrm{site}}}\left(\sqrt{{\left(z_{⊥}+v\right)}^{2}+4x_{\|}}+\left(z_{⊥}+v\right)\right).$$

Now we see that the quantity inside the large parentheses is $2Z_{\mathrm{site}}$, and so the mean site occupancy reduces to

(163) $$n_{⊥}=\frac{z_{⊥}}{\sqrt{{\left(z_{⊥}+v\right)}^{2}+4x_{\|}}}.$$

The same steps lead to the mean site occupancy for the vacancies *v*, which is

(164) $$n_{v}=\frac{v}{\sqrt{{\left(z_{⊥}+v\right)}^{2}+4x_{\|}}}.$$

Since the parallel dimers $x_{\|}$ each occupy two sites, the mean occupancy per site must be doubled, and we have the mean site occupancy for the parallel dimers as

(165) $$n_{\|}=2\frac{1}{\beta Z_{\mathrm{site}}}\frac{\partial Z_{\mathrm{site}}}{\partial \mu_{\|}}=\frac{2}{2\beta Z_{\mathrm{site}}}\frac{\partial }{\partial \mu_{\|}}\left(z_{⊥}+v+\sqrt{{\left(z_{⊥}+v\right)}^{2}+4x_{\|}}\right).$$

Taking the derivative, we have

(166) $$n_{\|}=\frac{1}{\beta Z_{\mathrm{site}}}\frac{1}{2}{\left[{\left(z_{⊥}+v\right)}^{2}+4x_{\|}\right]}^{-\frac{1}{2}}\left(4\beta x_{\|}\right).$$

Simplifying yields

(167) $$n_{\|}=\frac{2x_{\|}}{Z_{\mathrm{site}}\sqrt{{\left(z_{⊥}+v\right)}^{2}+4x_{\|}}}.$$

The sum of the occupancies for the three species should be 1, and so let us check that. The sum is

(168) $$n_{⊥}+n_{\|}+n_{v}=\frac{\left[\left(z_{⊥}+v\right)Z_{\mathrm{site}}+2x_{\|}\right]}{Z_{\mathrm{site}}\sqrt{{\left(z_{⊥}+v\right)}^{2}+4x_{\|}}}.$$

Substituting $Z_{\mathrm{site}}$ in the numerator, we have

(169) $$n_{⊥}+n_{\|}+n_{v}=\frac{1}{Z_{\mathrm{site}}\sqrt{{\left(z_{⊥}+v\right)}^{2}+4x_{\|}}}\left\{\frac{1}{2}\left(z_{⊥}+v\right)\left[\left(z_{⊥}+v\right)+\sqrt{{\left(z_{⊥}+v\right)}^{2}+4x_{\|}}\right]+2x_{\|}\right\}.$$

Factoring $\frac{1}{2}$ from the numerator and expanding, we have

(170) $$n_{⊥}+n_{\|}+n_{v}=\frac{1}{2Z_{\mathrm{site}}\sqrt{{\left(z_{⊥}+v\right)}^{2}+4x_{\|}}}\left[{\left(z_{⊥}+v\right)}^{2}+4x_{\|}+\left(z_{⊥}+v\right)\sqrt{{\left(z_{⊥}+v\right)}^{2}+4x_{\|}}\right].$$

Factoring $\sqrt{{\left(z_{⊥}+v\right)}^{2}+4x_{\|}}$ from the numerator produces

(171) $$n_{⊥}+n_{\|}+n_{v}=\frac{\sqrt{{\left(z_{⊥}+v\right)}^{2}+4x_{\|}}}{2Z_{\mathrm{site}}\sqrt{{\left(z_{⊥}+v\right)}^{2}+4x_{\|}}}\left[\sqrt{{\left(z_{⊥}+v\right)}^{2}+4x_{\|}}+\left(z_{⊥}+v\right)\right].$$

The last factor on the right is exactly $2Z_{\mathrm{site}}$, and so the right-hand side reduces to one, and we have

(172) $$n_{⊥}+n_{\|}+n_{v}=1.$$

Therefore, the total occupancy is, correctly, unity.

## 11. Results for the Entropy, Average Occupation, and Total Charge

Under certain conditions, DNA molecules condense. In this process, the DNA chain rolls into a tight toroid. If a DNA strand were simply placed in aqueous solution, this would not happen, because the phosphate groups that form the DNA backbone are negative, so that one length of the chain repels another. However, in a solution containing cations, the positive cations are electrostatically attracted to the negative backbone, neutralizing it or even inverting the charge. When the charge is inverted, the DNA chain dressed by the cations becomes positive, allowing it to coil up compactly.

Understanding the statistical mechanics of such processes gives insight into the evolution of complex life and the dynamics that maintains it. This led two of the authors to use methods borrowed from the physics of interacting particles and quantum field theory to the electrostatics of biomolecules [9,10] using lattice gas models. Such models regard all ions, monomers, dimers, or larger polyions as pointlike particles, completely abandoning the geometric constraints that come from the extended nature of the adsorbing species. All the geometry inherent in attaching a polyion flat against a surface so that it covers several lattice sites is lost.

In the plots of this section, any numerical parameters used were taken from our earlier work [9,10]. In these plots, the horizontal axis is the dimensionless product $\beta \varepsilon_{\|}$. Because parallel dimers occupy two sites, while the perpendicular dimers only occupy one, we set $\beta \varepsilon_{⊥}=\frac{1}{2}\beta \varepsilon_{\|}$. Strong binding to the lattice occurs when $\beta \varepsilon_{\|}$ is large and negative, while the weak binding region is where $\beta \varepsilon_{\|}$ is positive. Assuming that the DNA double helix is in equilibrium with a solution of dimers, all the chemical potentials must be the same and be equal to the chemical potential of dimers in solution given by $\beta \mu_{\mathrm{dimer}}$. The plots are all for the single physiological temperature of T≃310 K, with $\beta \mu_{\mathrm{dimer}}≃0.79$.

The Fisher dimer model, as formulated in the previous sections, describes parallel dimers, with activity $x_{\|}$, perpendicular dimers, with activity $z_{⊥}$, and vacancies with activity *v*. These vacancies are on the negative backbone, and each of these vacant sites carries a charge of −1, in units of the magnitude of the electron charge. The dimers have charge +2, with one positive charge on each end. Then, electrostatic attraction causes the dimers to bind to the vacant sites. They can do so in two ways, first by lying flat, parallel to the DNA chain, and binding to two vacant sites, so that they have an activity of $x_{\|}$. Alternatively, they can protrude at right angles to the DNA chain, with only one end bound to the chain and activity $z_{⊥}$. These two possibilities are called parallel and perpendicular dimers, with the perpendicular dimers acting as the monomers in the dimer, monomer, and vacancy model.

**Figure 16:**
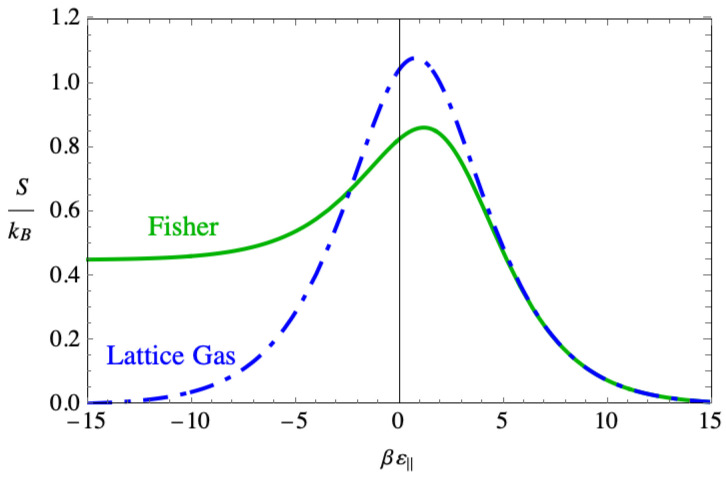
The plot of the two entropies given by the lattice gas model and the Fisher model of dimers.

Figure 16 shows the entropies given by the Fisher dimer model and by the non-interacting lattice gas model. The striking feature is the way that entropy appears to plateau in the strong binding region. The lattice gas model does not show this because all the dimers lie flat on the lattice by $\beta \varepsilon_{\|}∼-10$. The nonzero value of the entropy in the Fisher dimer model suggests that disorder persists in this region even as the binding force becomes quite strong.

**Figure 17:**
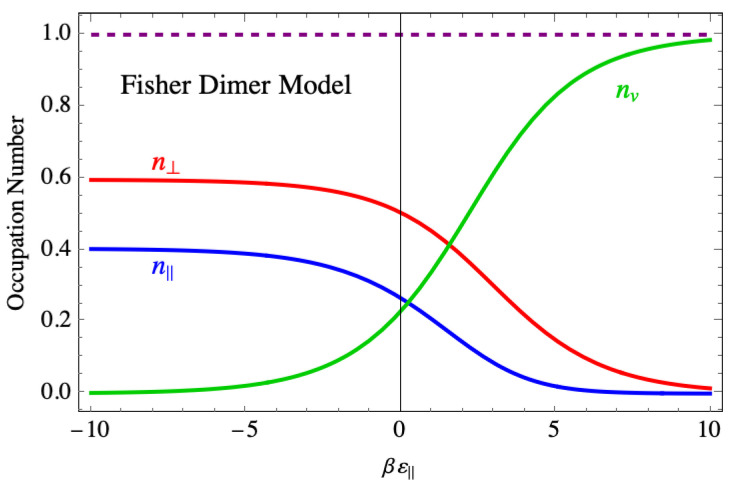
The average occupation of species in the Fisher model of dimers showing species mixing even at large negative binding energy. The purple dashed line is a numerical check showing that the sum of occupancies is one, as demonstrated analytically in the text.

To understand this disorder in more detail, Figure 17 shows the site occupancies for the Fisher dimer model. In the region of strong binding, both the site occupancies for the parallel and perpendicular dimers also plateau, even though the number of vacancies drops to near zero. This indicates that the disorder that leads to the nonvanishing entropy consists of a mixture of parallel and perpendicular dimers, occupying practically all the sites and accounting for the nonzero value of the entropy as the binding force becomes strong.

**Figure 18:**
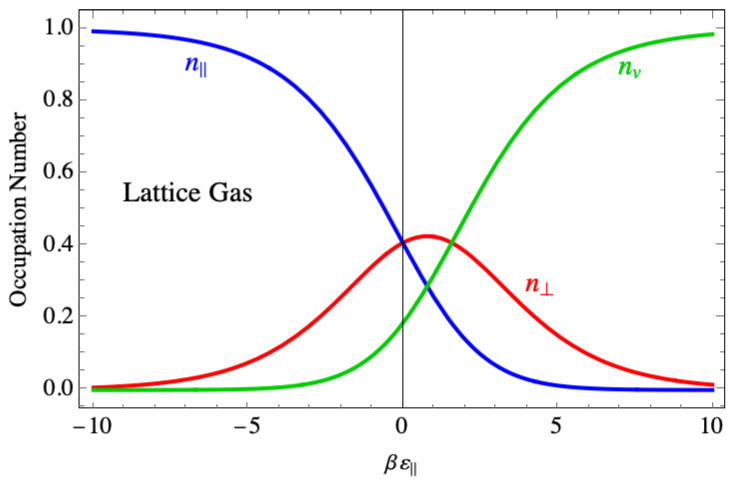
The average occupation of species in the Fisher model of dimers showing species mixing even at large negative binding energy.

For comparison, Figure 18 shows the corresponding site occupancies for the lattice gas model. There, the perpendicular dimer occupancy drops to zero in the region where the binding force is strong, as does the vacancy density, and the lattice is completely occupied by parallel dimers. An interpretation of the contrasting behavior between the two models is that the actual shape of a dimer spanning two sites when it lies parallel is responsible for the disorder, while in the lattice gas model, the parallel dimers are treated as though they are monomers, but with twice the binding energy.

**Figure 19:**
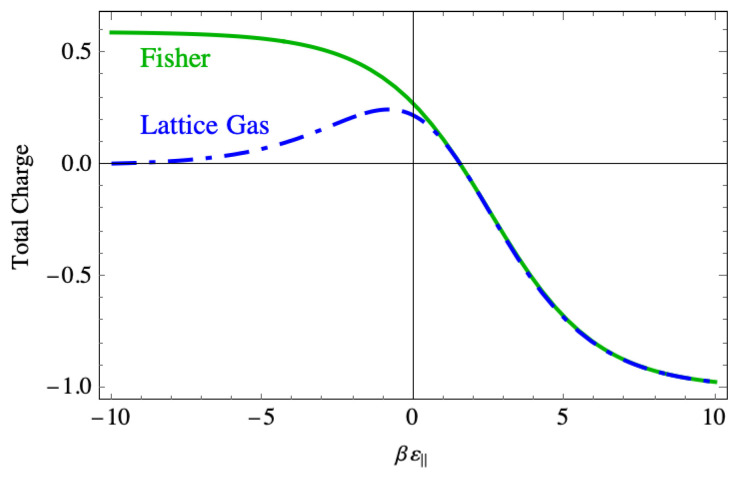
A comparison of the total charge vs. the binding energy on the lattice between the lattice gas model and the Fisher model.

The phenomenon of the DNA strand rolling up compactly requires excess charge, and so Figure 19 shows the total charge $n_{⊥}+n_{v}$. This charge is positive when the binding force is strong because in that region there are few negative vacancies, but still a large number of perpendicular dimers, while each parallel dimer neutralizes two sites. That persistent positive charge in the Fisher model does not appear in the lattice gas model, where the charge drops to near zero in that region. In the weak binding region, the charge becomes small in the same way in both models, and the two curves lie on top of one another.

In the context of DNA compaction, this excess charge is the physically most important feature seen from the Fisher model. This is because it leads to larger and more persistent charge inversion than is seen in the lattice gas model. It does so because once a sequence of parallel dimer–vacancy–parallel dimer forms, the only way the vacancy can be filled is with a perpendicular dimer. This must be the persistent disorder that occurs in the region of strong binding force.

## 12. Discussion

We have described a simplified version of Fisher’s derivation of the partition function of the completely filled ν=1 dimer model. This provides the entropy as calculated by Fisher, and the mathematics described has some relevance to the Ising spin model. While this work was in progress, we became aware of the paper by Allegra and Fortin [17] that proposes the use of Grassmann variables to solve the d=2 dimer problem including monomers, obtaining the partition function as a product of two Pfaffians. That opens the way for entropy and site occupancy calculations for the two-dimensional sheet following methods described here.

After extracting the partition function for a one-dimensional dimer–monomer–vacancy system from that for a ν=1 two-leg ladder, we compared the entropy and site occupations as a function of the binding energy to the lattice with the similar results for the lattice gas. The entropy appeared to plateau at a nonzero value in the strong-binding-force region, suggesting that disorder persists in that region.

In this context, the dimers represent dimeric polyions with a unit positive charge on each end that neutralizes the unit negative charge on the backbone lattice site, so the parallel dimers are effectively charge-neutral. The monomers represent the same dimers but oriented perpendicular to the surface so that only one end neutralizes the backbone lattice site to which it is attached, and it makes a unit-positive-charge contribution to the charge density on the chain, while each vacant site contributes its unit negative charge. In the strong-binding-force region, most lattice sites are occupied and few vacancies remain, but the perpendicular dimers scattered among the parallel dimers is a source of disorder. This leads to the persistent excess charge in the strong-binding region, and provides the disorder that accounts for the persistent plateau in the entropy there as well.

In our earlier paper [10] using the lattice gas model, we included electrostatic repulsion among the dimers, monomers, and negative empty sites on the DNA backbone to one-loop order. That did produce a reduction in the entropy by up to 1/3 of the total, but only in the weak-binding region of the plot. Consequently, this reduction would not affect the strong-binding region where the dimer model entropy appears to plateau at a nonzero value and the excess positive charge persists. Because this strong-binding region is the more important one, the geometry of the extended dimer occupying more than one site is the more important physically.

In other words, our conclusion from this work is that if physical consequences are the concern, the extended geometry of the attached molecules has more influence than the electrostatic many-particle interactions of species that carry electrical charge. When determining entropy, the shape of the attached molecule and the volume it excludes does matter, and ways of including these effects are needed.

## Data Availability

There is no data involved. This is a theoretical study.

## Appendix A. Trace Theorems of the *A^μ^* Matrices

We now prove the trace theorems that we need in order to study the statistical mechanics of the ν=1 dimer model on a square lattice. The commutation law with ν=μ shows that

(A1) $$2A^{\mu }A^{\nu }=2g^{\mu \mu }=2I_{d}.$$

This ensures that the trace of a single $A^{\mu }$ matrix vanishes, because inserting ${\left(A^{\nu }\right)}^{2}=I_{d}$ and using the cyclic property of the trace yields

(A2) $$\mathrm{Tr}\left(A^{\mu }\right)=\mathrm{Tr}\left[A^{\mu }{\left(A^{\nu }\right)}^{2}\right]=\mathrm{Tr}\left(A^{\nu }A^{\mu }A^{\nu }\right)=\mathrm{Tr}\left(-A^{\mu }A^{\nu }A^{\nu }\right),$$

where to obtain the third equality we anticommuted $A^{\nu }$ and $A^{\mu }$, introducing a minus sign. Now, the product $-A^{\mu }A^{\nu }A^{\nu }$ can be regarded as some *d*-dimensional matrix *M* with matrix elements $m_{ij}$. Then, $\mathrm{Tr}\left(-M\right)=\sum_{j=1}^{d}\left(-m_{jj}\right)=-\sum_{j=1}^{d}m_{jj}=-\mathrm{Tr}\left(M\right)$. Thus

(A3) $$\mathrm{Tr}\left(A^{\mu }\right)=\mathrm{Tr}\left(-A^{\mu }A^{\nu }A^{\nu }\right)=-\mathrm{Tr}\left(A^{\mu }A^{\nu }A^{\nu }\right)=-\mathrm{Tr}\left(A^{\mu }\right).$$

This can only be true if

(A4) $$\mathrm{Tr}(A^{\mu })=0.$$

This is easily generalized to the trace of any product of an odd number of $A_{j}$ matrices, because then

(A5) $$\begin{array}{ccc} \mathrm{Tr}(A^{\mu_{1}}A^{\mu_{2}}\cdots A^{\mu_{2n+1}}) & = & \mathrm{Tr}\left[A^{\mu_{1}}A^{\mu_{2}}\cdots A^{\mu_{2n+1}}{\left(A^{\nu }\right)}^{2}\right]=\mathrm{Tr}\left(A^{\nu }A^{\mu_{1}}A^{\mu_{2}}\cdots A^{\mu_{2n+1}}A^{\nu }\right)\\ & = & \mathrm{Tr}[{\left(-1\right)}^{2n+1}A^{\mu_{1}}A^{\mu_{2}}\cdots A^{\mu_{2n+1}}A^{\nu }A^{\nu }], \end{array}$$

since an odd number of interchanges is required to move $A^{\nu }$ past 2n+1 other matrices. We have

(A6) $$\mathrm{Tr}\left(A^{\mu_{1}}A^{\mu_{2}}\cdots A^{\mu_{2n+1}}\right)={\left(-1\right)}^{2n+1}\mathrm{Tr}\left(A^{\mu_{1}}A^{\mu_{2}}\cdots A^{\mu_{2n+1}}\right)=-\mathrm{Tr}\left(A^{\mu_{1}}A^{\mu_{2}}\cdots A^{\mu_{2n+1}}\right),$$

so that the trace is zero,

(A7) $$\mathrm{Tr}(A^{\mu_{1}}A^{\mu_{2}}\cdots A^{\mu_{2n+1}})=0.$$

However, the trace of the product of an even number of $A^{\mu }$ matrices does not always vanish. The simplest example is

(A8) $$A^{\mu }A^{\nu }=2g^{\mu \nu }-A^{\nu }A^{\mu }.$$

Taking the trace gives

(A9) $$\mathrm{Tr}\left(A^{\mu }A^{\nu }\right)=2\mathrm{Tr}\left(g^{\mu \nu }\right)+\mathrm{Tr}\left(-A^{\nu }A^{\mu }\right)=2\mathrm{Tr}\left(g^{\mu \nu }\right)-\mathrm{Tr}\left(A^{\nu }A^{\mu }\right)=2\mathrm{Tr}\left(g^{\mu \nu }\right)-\mathrm{Tr}\left(A^{\mu }A^{\nu }\right),$$

where in the last equality we used the cyclic property of the trace. Since the last trace on the right is the same as the expression we started with, moving the last term on the right to the left side gives

(A10) $$\mathrm{Tr}\left(A^{\mu }A^{\nu }\right)=\mathrm{Tr}\left(g^{\mu \nu }\right),$$

after canceling a factor of two. When $\nu =\mu ,\mathrm{Tr}\left(g^{\mu \nu }\right)=\mathrm{Tr}\left(g^{\mu \mu }\right)=d$.

Now, let us tackle the general case of arbitrary numbers of both matched pairs and unmatched $A^{\mu }$s. Any product can be reduced to sums of products of the $g^{\mu \nu }$ and a leftover term by repeated application of the anticommutation relation $A^{\mu }A^{\nu }=2g^{\mu \nu }-A^{\nu }A^{\mu }$. Consider

(A11) $$\begin{array}{ccc} A^{\mu_{1}}A^{\mu_{2}}\cdots A^{\mu_{n}} & = & \left(2g^{\mu_{1}\mu_{2}}-A^{\mu_{2}}A^{\mu_{1}}\right)A^{\mu_{3}}A^{\mu_{4}}\cdots A^{\mu_{n}}\\ & = & 2g^{\mu_{1}\mu_{2}}A^{\mu_{3}}A^{\mu_{4}}\cdots A^{\mu_{n}}-A^{\mu_{2}}A^{\mu_{1}}A^{\mu_{3}}A^{\mu_{4}}\cdots A^{\mu_{n}}. \end{array}$$

Continuing to move $A^{\mu_{1}}$ to the end in the last term gives

(A12) $$\begin{array}{ccc} A^{\mu_{1}}A^{\mu_{2}}\cdots A^{\mu_{n}} & = & 2g^{\mu_{1}\mu_{2}}A^{\mu_{3}}A^{\mu_{4}}\cdots A^{\mu_{n}}-A^{\mu_{2}}\left(2g^{\mu_{1}\mu_{3}}-A^{\mu_{3}}A^{\mu_{1}}\right)A^{\mu_{4}}A^{\mu_{5}}\cdots A^{\mu_{n}}\\ & = & 2g^{\mu_{1}\mu_{2}}A^{\mu_{3}}A^{\mu_{4}}\cdots A^{\mu_{n}}-A^{\mu_{2}}\left(2g^{\mu_{1}\mu_{3}}\right)A^{\mu_{4}}A^{\mu_{5}}\cdots A^{\mu_{n}}\\ & & +A^{\mu_{2}}A^{\mu_{3}}A^{\mu_{1}}A^{\mu_{4}}A^{\mu_{5}}\cdots A^{\mu_{n}}. \end{array}$$

The metric $g^{\mu \nu }$ commutes with all the $A^{\mu }$, and can be moved to the front of each term. We then have the general pattern

(A13) $$\begin{array}{ccc} A^{\mu_{1}}A^{\mu_{2}}\cdots A^{\mu_{n}} & = & 2g^{\mu_{1}\mu_{2}}A^{\mu_{3}}A^{\mu_{4}}\cdots A^{\mu_{n}}-2g^{\mu_{1}\mu_{3}}A^{\mu_{2}}A^{\mu_{4}}A^{\mu_{5}}\cdots A^{\mu_{n}}\\ & & +2g^{\mu_{1}\mu_{4}}A^{\mu_{2}}A^{\mu_{4}}A^{\mu_{5}}\cdots A^{\mu_{n}}-\cdots \\ & & +{\left(-1\right)}^{n-1}A^{\mu_{2}}A^{\mu_{3}}A^{\mu_{4}}A^{\mu_{5}}\cdots A^{\mu_{n}}A^{\mu_{1}}, \end{array}$$

with the signs alternating from one term to the next, and the last having an overall parity factor of ${\left(-1\right)}^{n-1}$ because n−1 interchanges are needed to move $A^{\mu_{1}}$ to the end.

Although we could now apply this general formula, which we will call the contraction theorem, to continue to reduce the sub-products like $A^{\mu_{3}}A^{\mu_{4}}\cdots A^{\mu_{n}}$, let us first take the trace of this expression and use the cyclic property to see that

(A14) $$\begin{array}{ccc} \mathrm{Tr}(A^{\mu_{1}}A^{\mu_{2}}\cdots A^{\mu_{n}}) & = & 2\mathrm{Tr}\left(g^{\mu_{1}\mu_{2}}A^{\mu_{3}}A^{\mu_{4}}\cdots A^{\mu_{n}}\right)-2\mathrm{Tr}\left(g^{\mu_{1}\mu_{3}}A^{\mu_{2}}A^{\mu_{4}}A^{\mu_{5}}\cdots A^{\mu_{n}}\right)\\ & & +2\mathrm{Tr}(g^{\mu_{1}\mu_{4}}A^{\mu_{2}}A^{\mu_{3}}A^{\mu_{5}}\cdots A^{\mu_{n}})-\cdots \\ & & +{\left(-1\right)}^{n-1}\mathrm{Tr}\left(A^{\mu_{1}}A^{\mu_{2}}A^{\mu_{3}}A^{\mu_{4}}A^{\mu_{5}}\cdots A^{\mu_{n}}\right). \end{array}$$

Here, the trace on the left is the same as the last trace on the right, and if *n* is even, they are added to give

(A15) $$\begin{array}{ccc} \mathrm{Tr}(A^{\mu_{1}}A^{\mu_{2}}\cdots A^{\mu_{n}}) & = & \mathrm{Tr}\left(g^{\mu_{1}\mu_{2}}A^{\mu_{3}}A^{\mu_{4}}\cdots A^{\mu_{n}}\right)-\mathrm{Tr}\left(g^{\mu_{1}\mu_{3}}A^{\mu_{2}}A^{\mu_{4}}A^{\mu_{5}}\cdots A^{\mu_{n}}\right)\\ & & +\mathrm{Tr}(g^{\mu_{1}\mu_{4}}A^{\mu_{2}}A^{\mu_{3}}A^{\mu_{5}}\cdots A^{\mu_{n}})-\cdots . \end{array}$$

However, if *n* is odd, they cancel, and the sum of all terms remaining on the right must vanish,

(A16) $$\begin{array}{ccc} \mathrm{Tr}(g^{\mu_{1}\mu_{2}}A^{\mu_{3}}A^{\mu_{4}}\cdots A^{\mu_{n}}) & - & \mathrm{Tr}(g^{\mu_{1}\mu_{3}}A^{\mu_{2}}A^{\mu_{4}}A^{\mu_{5}}\cdots A^{\mu_{n}})\\ & + & \mathrm{Tr}(g^{\mu_{1}\mu_{4}}A^{\mu_{2}}A^{\mu_{3}}A^{\mu_{5}}\cdots A^{\mu_{n}})-\cdots =0. \end{array}$$

A second consequence of *n* being odd is that it is not possible to continue the reduction to all possible pairs, that is, a product of $g^{\mu \nu }$. There will always be one $A^{\mu }$ left over. In that case, since $g^{\mu \nu }=\delta^{\mu \nu }I_{d}$, the trace becomes either zero if at least one factor of $g^{\mu \nu }$ vanishes, or we have just $\mathrm{Tr}(A^{\mu })=0$. Consequently, the trace always vanishes if *n* is odd.

For *n* even, we have

(A17) $$\begin{array}{ccc} \mathrm{Tr}(A^{\mu_{1}}A^{\mu_{2}}\cdots A^{\mu_{n}}) & = & \mathrm{Tr}\left(g^{\mu_{1}\mu_{2}}A^{\mu_{3}}A^{\mu_{4}}\cdots A^{\mu_{n}}\right)-\mathrm{Tr}\left(g^{\mu_{1}\mu_{3}}A^{\mu_{2}}A^{\mu_{4}}A^{\mu_{5}}\cdots A^{\mu_{n}}\right)\\ & & +\mathrm{Tr}(g^{\mu_{1}\mu_{4}}A^{\mu_{2}}A^{\mu_{3}}A^{\mu_{5}}\cdots A^{\mu_{n}})-\cdots \\ & = & \sum_{k=2}^{n}{\left(-1\right)}^{k}\mathrm{Tr}\left(g^{\mu_{1}\mu_{k}}\prod_{\begin{array}{c} l=2\\ l\neq k \end{array}}^{n}A^{\mu_{l}}\right), \end{array}$$

because $g^{\mu \nu }=\delta^{\mu \nu }I_{d}$ picks out the term or terms in the sum over *k* that have $\mu_{k}=\mu_{1}$ and replaces the factor $g^{\mu_{1}\mu_{k}}$ by the *d*-dimensional identity, which can be omitted from the remaining product. The simplest case is when only one term has $\mu_{k}=\mu_{1}$, and that is the only possibility that we will encounter, because the construction we will use can only place at most two $A^{\mu }$ on a single link, or dual site, of the lattice. Then, the trace above reduces to

(A18) $$\mathrm{Tr}\left(A^{\mu_{1}}A^{\mu_{2}}\cdots A^{\mu_{n}}\right)={\left(-1\right)}^{P}\mathrm{Tr}\left(\prod_{l=1}^{n-2}A^{\mu_{l}}\right),$$

with *P* the appropriate permutation index. Then the contraction theorem can be applied again to the remaining product. Continuing the process eventually reduces the right side to a permutation factor ${\left(-1\right)}^{P}$ times the trace of $I_{d}$, which is *d*, or to a product of unmatched $A^{\mu }$, the trace of which vanishes.

## Appendix B. Pfaffians and Determinants

Pfaffians are rarely discussed. They do not appear in common mathematical physics textbooks, but they have shown up in the physics literature—there is a Pfaffian quantum Hall state, for example [18,19].

A Pfaffian is similar to a determinant, but it is taken on the upper right triangle of an antisymmetric matrix. One of the Pfaffian’s most important properties is that its square is equal to the determinant of the corresponding antisymmetric matrix *M*,

(A19) $${\left(\mathrm{Pf}\left[M\right]\right)}^{2}=\det\left[M\right],$$

where an example of dimension 2h is the antisymmetric matrix

(A20) $$M=\left(\begin{array}{cccccc} 0 & a_{1,2} & a_{1,3} & a_{1,4} & \cdots & a_{1,2h}\\ -a_{1,2} & 0 & a_{2,3} & a_{2,4} & \cdots & a_{2,2h}\\ -a_{1,3} & -a_{2,3} & 0 & a_{3,4} & \cdots & a_{3,2h}\\ -a_{1,4} & -a_{2,4} & -a_{3,4} & 0 & \ddots & \vdots \\ \vdots & \vdots & \vdots & \ddots & \ddots & a_{2h-1,2h}\\ -a_{1,2h} & -a_{2,2h} & -a_{3,2h} & \cdots & -a_{2h-1,2h} & 0 \end{array}\right).$$

The corresponding Pfaffian is sometimes written as

(A21) $$\mathrm{Pf}\left[M\right]=\left.\begin{array}{cccccc} & |a_{1,2} & a_{1,3} & a_{1,4} & \cdots & a_{1,2h}\\ & & a_{2,3} & a_{2,4} & \cdots & a_{2,2h}\\ & & & a_{3,4} & \cdots & a_{3,2h}\\ & & & & \ddots & \vdots \\ & & & & & a_{2h-1,2h} \end{array}\right|.$$

Here, we use the convention that *M* is an antisymmetric matrix, Det[M] is its determinant, and Pf[M] is the Pfaffian of the upper right triangle of *M* (with the diagonal, which consists only of zeros, deleted).

The Pfaffian expansion by the first row is like a regular determinant with the notable caveat that both the *r*th row and column and the *s*th row and column are deleted. Suppose that we illustrate with an example of order 2h=6,

(A22) $$\mathrm{Pf}\left[M\right]=\left.\begin{array}{cccccc} & |a_{1,2} & a_{1,3} & a_{1,4} & a_{1,5} & a_{1,6}\\ & & a_{2,3} & a_{2,4} & a_{2,5} & a_{2,6}\\ & & & a_{3,4} & a_{3,5} & a_{3,6}\\ & & & & a_{4,5} & a_{4,6}\\ & & & & & a_{5,6} \end{array}\right|.$$

Expanding by the top row gives a first term of

(A23) $$\mathrm{Pf}\left[M\right]=a_{1,2}\times \left.\begin{array}{ccc} |a_{3,4} & a_{3,5} & a_{3,6}\\ & a_{4,5} & a_{4,6}\\ & & a_{5,6} \end{array}\right|+\cdots .$$

Including the remaining terms, which have alternating signs just like a determinant, we have

(A24) $$\begin{array}{ccc} \mathrm{Pf}[M] & = & a_{1,2}\times \left.\begin{array}{ccc} |a_{3,4} & a_{3,5} & a_{3,6}\\ & a_{4,5} & a_{4,6}\\ & & a_{5,6} \end{array}\right|-a_{1,3}\times \left.\begin{array}{ccc} |a_{2,4} & a_{2,5} & a_{2,6}\\ & a_{4,5} & a_{4,6}\\ & & a_{5,6} \end{array}\right|\\ & + & a_{1,4}\times \left.\begin{array}{ccc} |a_{2,3} & a_{2,5} & a_{2,6}\\ & a_{3,5} & a_{3,6}\\ & & a_{5,6} \end{array}\right|-a_{1,5}\times \left.\begin{array}{ccc} |a_{2,3} & a_{2,4} & a_{2,6}\\ & a_{3,4} & a_{3,6}\\ & & a_{4,6} \end{array}\right|\\ & + & a_{16}\times \left.\begin{array}{ccc} |a_{2,3} & a_{2,4} & a_{2,5}\\ & a_{3,4} & a_{3,5}\\ & & a_{4,5} \end{array}\right|. \end{array}$$

Continuing, we expand each of these minors by its first row. The first gives

(A25) $$\left.\begin{array}{ccc} |a_{3,4} & a_{3,5} & a_{3,6}\\ & a_{4,5} & a_{4,6}\\ & & a_{5,6} \end{array}\right|=a_{3,4}\times \left.\begin{array}{cc} |a_{4,5} & a_{4,6}\\ & a_{5,6} \end{array}\right|=a_{3,4}a_{5,6}.$$

Including the rest of the terms, the Pfaffian is

(A26) $$\begin{array}{ccc} \mathrm{Pf}[M] & = & a_{1,2}\left(a_{3,4}a_{5,6}-a_{3,5}a_{4,6}+a_{3,6}a_{4,5}\right)-a_{1,3}\left(a_{2,4}a_{5,6}-a_{2,5}a_{4,6}+a_{2,6}a_{4,5}\right)\\ & & +a_{1,4}\left(a_{2,3}a_{5,6}-a_{2,5}a_{3,6}+a_{2,6}a_{3,5}\right)-a_{1,5}\left(a_{2,3}a_{4,6}-a_{2,4}a_{3,6}+a_{2,6}a_{3,4}\right)\\ & & +a_{1,6}\left(a_{2,3}a_{4,5}-a_{2,4}a_{3,5}+a_{2,5}a_{3,4}\right)\\ & = & a_{1,2}a_{3,4}a_{5,6}-a_{1,2}a_{3,5}a_{4,6}+a_{1,2}a_{3,6}a_{4,5}-a_{1,3}a_{2,4}a_{5,6}+a_{1,3}a_{2,5}a_{4,6}\\ & & -a_{1,3}a_{2,6}a_{4,5}+a_{1,4}a_{2,3}a_{5,6}-a_{1,4}a_{2,5}a_{3,6}+a_{1,4}a_{2,6}a_{3,5}-a_{1,5}a_{2,3}a_{4,6}\\ & & +a_{1,5}a_{2,4}a_{3,6}-a_{1,5}a_{2,6}a_{3,4}+a_{1,6}a_{2,3}a_{4,5}-a_{1,6}a_{2,4}a_{3,5}+a_{1,6}a_{2,5}a_{3,4}. \end{array}$$

This is the full expansion of the Pfaffian.

## Appendix C. Block by Block Multiplication

In this context, it is important to realize that matrices can be multiplied block by block. Let us justify this using a simple example. Suppose that we have a 2n×2n matrix *A* divided into four n×n blocks, $A^{\left(1,1\right)}$, $A^{\left(1,2\right)}$, $A^{\left(2,1\right)}$, and $A^{\left(2,2\right)}$,

(A27) $$A=\left(\begin{array}{cc} A^{\left(1,1\right)} & A^{\left(1,2\right)}\\ A^{\left(2,1\right)} & A^{\left(2,2\right)} \end{array}\right),$$

and a similar division of a second 2n×2n matrix *B* divided similarly,

(A28) $$B=\left(\begin{array}{cc} B^{\left(1,1\right)} & B^{\left(1,2\right)}\\ B^{\left(2,1\right)} & B^{\left(2,2\right)} \end{array}\right).$$

The matrix elements of the product of these two matrices are

(A29) $${\left[AB\right]}_{ik}=\sum_{j=1}^{2n}A_{ij}B_{jk}.$$

The sum over *j* can be divided up into two sums,

(A30) $${\left[AB\right]}_{ik}=\sum_{j=1}^{n}A_{ij}B_{jk}+\sum_{j=n+1}^{2n}A_{ij}B_{jk}.$$

Suppose that 1≤i≤n and 1≤k≤n. This gives the upper left entry in the block product. The matrix elements that appear in the product are

(A31) $${\left[AB\right]}_{ik}=\sum_{j=1}^{n}{\left[A^{\left(1,1\right)}\right]}_{ij}{\left[B^{\left(1,1\right)}\right]}_{jk}+\sum_{j=n+1}^{2n}{\left[A^{\left(1,2\right)}\right]}_{ij}{\left[B^{\left(2,1\right)}\right]}_{jk}=A^{\left(1,1\right)}B^{\left(1,1\right)}+A^{\left(1,2\right)}B^{\left(2,1\right)},$$

where we wrote the result as a block-matrix product in the last equality. If n+1≤i≤2n and 1≤k≤n, we obtain the lower left entry in the block product. The matrix elements that appear in the sum are

(A32) $${\left[AB\right]}_{ik}=\sum_{j=1}^{n}{\left[A^{\left(1,1\right)}\right]}_{ij}{\left[B^{\left(1,2\right)}\right]}_{jk}+\sum_{j=n+1}^{2n}{\left[A^{\left(1,2\right)}\right]}_{ij}{\left[B^{\left(2,2\right)}\right]}_{jk}=A^{\left(1,1\right)}B^{\left(1,2\right)}+A^{\left(1,2\right)}B^{\left(2,2\right)}.$$

If n+1≤i≤2n and n+1≤k≤2n, we obtain the lower right entry in the block product. The matrix elements that appear in the sum are

(A33) $${\left[AB\right]}_{ik}=\sum_{j=1}^{n}{\left[A^{\left(2,1\right)}\right]}_{ij}{\left[B^{\left(1,2\right)}\right]}_{jk}+\sum_{j=n+1}^{2n}{\left[A^{\left(2,2\right)}\right]}_{ij}{\left[B^{\left(2,2\right)}\right]}_{jk}=A^{\left(2,1\right)}B^{\left(1,2\right)}+A^{\left(2,2\right)}B^{\left(2,2\right)}.$$

Now, if we calculate the product by merely using block indices, we obtain

(A34) $$AB=\left(\begin{array}{cc} \left[A^{\left(1,1\right)}B^{\left(1,1\right)}+A^{\left(1,2\right)}B^{\left(2,1\right)}\right] & \left[A^{\left(1,1\right)}B^{\left(1,2\right)}+A^{\left(2,1\right)}B^{\left(2,2\right)}\right]\\ {}[A^{\left(2,1\right)}B^{\left(1,1\right)}+A^{\left(2,2\right)}B^{\left(2,1\right)}] & \left[A^{\left(2,1\right)}B^{\left(1,2\right)}+A^{\left(2,2\right)}B^{\left(2,2\right)}\right] \end{array}\right),$$

where we offset the individual blocks using brackets. This is the same result. The conclusion is that block matrices can be multiplied block by block.

## Appendix D. Evaluation of *t*_*ω*,*ω*′_

This is a derivation of the matrix element of the block *Y* in antidiagonal form.

(A35) $$\begin{array}{ccc} t_{\omega ,\omega^{\prime }}=\sum_{k=1}^{n_{c}}v_{k}^{\omega *}v_{n_{c}-k+1}^{\omega^{\prime }} & = & \frac{2}{n+1}\sum_{k=1}^{n_{c}}\left[\sqrt{\frac{2}{n_{c}+1}}{\left(-i\right)}^{k+1}\sin\left(\frac{\pi \omega k}{n_{c}+1}\right)\right]\\ & & \times \left[\sqrt{\frac{2}{n_{c}+1}}i^{n_{c}-k+2}\sin\left(\frac{\pi \omega^{\prime }\left(n_{c}-k+1\right)}{n_{c}+1}\right)\right]. \end{array}$$

Simplifying somewhat, the sum becomes

(A36) $$t_{\omega ,\omega^{\prime }}=\frac{2}{n_{c}+1}i^{n_{c}+3}\sum_{k=1}^{n_{c}}{\left(-1\right)}^{k+1}\sin\left(\frac{\pi \omega k}{n_{c}+1}\right)\sin\left(\frac{\pi \omega^{\prime }\left(n_{c}-k+1\right)}{n_{c}+1}\right).$$

The next task is to evaluate the sum of the product of sines

(A37) $$S=2\sum_{k=1}^{n_{c}}{\left(-1\right)}^{k+1}\sin\left(\frac{\pi \omega k}{n_{c}+1}\right)\sin\left(\frac{\pi \omega^{\prime }\left(n_{c}+1-k\right)}{n_{c}+1}\right).$$

First, the product of sines should be turned into a sum of cosines via the identity $\sin u\sin v=\frac{1}{2}\left[\cos\left(u-v\right)-\cos\left(u+v\right)\right]$. This gives

(A38) $$S=\sum_{k=1}^{n_{c}}\left\{{\left(-1\right)}^{k+1}\left[\cos\left(\frac{\pi \omega k}{n_{c}+1}-\frac{\pi \omega^{\prime }\left(n_{c}+1-k\right)}{n_{c}+1}\right)-\cos\left(\frac{\pi \omega k}{n_{c}+1}+\frac{\pi \omega^{\prime }\left(n_{c}+1-k\right)}{n_{c}+1}\right)\right]\right\},$$

which can be put into exponential form as

(A39) S=Re∑k=1nc(−1)k+1ei(πωk−πω′(nc+1−k))nc+1−ei(πωk+πω′(nc+1−k))nc+1,

where Re indicates that the real part of the sum is required. Because $\omega^{\prime }$ is a positive integer, there is a factor of $e^{\pm i\pi \omega^{\prime }}={\left(-1\right)}^{\omega^{\prime }}$ in each exponential. We also notice that each term can be written as an exponential to the $k^{\mathrm{th}}$ power, and we have

(A40) S=(−1)ω′+1Re∑k=1nc−eiπ(ω+ω′)nc+1k−−eiπ(ω−ω′)nc+1k.

Each term is now in the form of a geometric series [20] and can be summed as

(A41) $$\sum_{k=1}^{n_{c}}r^{k}=r\sum_{k=1}^{n_{c}}r^{k-1}=r\left(\frac{r^{n_{c}}-1}{r-1}\right),$$

giving

S=(−1)ω′+1Re−eiπ(ω+ω′)nc+1−eiπ(ω+ω′)nc+1nc−1−eiπ(ω+ω′)nc+1−1−−eiπ(ω−ω′)nc+1−eiπ(ω−ω′)nc+1nc−1−eiπ(ω−ω′)nc+1−1.

Distributing the exponentials in each term will cause the first exponential in each numerator contain $\frac{n_{c}+1}{n_{c}+1}$ and reduce to simpler exponentials as

(A42) S=(−1)ω′+1Re−(−1)nc+1eiπ(ω+ω′)+eiπ(ω+ω′)nc+11+eiπ(ω+ω′)nc+1+(−1)nc+1eiπ(ω−ω′)+eiπ(ω−ω′)nc+11+eiπ(ω−ω′)nc+1.

By Euler’s Formula, the first term in each numerator reduces to ${\left(-1\right)}^{\omega +\omega^{\prime }+n+1}$ and ${\left(-1\right)}^{\omega -\omega^{\prime }+n+1}$, respectively, yielding

(A43) S=(−1)ω′+1Re−(−1)ω+ω′+nc+1+eiπ(ω+ω′)nc+11+eiπ(ω+ω′)nc+1+(−1)ω−ω′+nc+1+eiπ(ω−ω′)nc+11+eiπ(ω−ω′)nc+1.

The denominator of the first term in the brackets is discontinuous at $\omega +\omega^{\prime }=n_{c}+1$, and so this geometric expansion can not be solved for instances when $\omega +\omega^{\prime }=n_{c}+1$. Those instances will be discussed in Section 4.

Moving forward from here depends on whether the exponents of the sign terms inside the Re operation are odd or even. If $\omega +\omega^{\prime }$ is even then $\omega -\omega^{\prime }$ must also be even, so that both terms inside the square brackets have the same parity in the first term in their numerators. When $\omega +\omega^{\prime }+n+1$ and $\omega -\omega^{\prime }+n+1$ are both even, it is easy to see that S reduces to

(A44) S=(−1)ω′+1Re(−1+1)=0.

When $\omega +\omega^{\prime }+n+1$ and $\omega -\omega^{\prime }+n+1$ are both odd, the situation is more complicated. The first terms in each of the numerators equal −1, and the sum simplifies to the form

(A45) S=(−1)ω′+1Re−eiπ(ω+ω′)nc+1−1eiπ(ω+ω′)nc+1+1+eiπ(ω−ω′)nc+1−1eiπ(ω−ω′)nc+1+1.

From here, the numerators and denominators are close to being sines and cosines. The factor $e^{-\frac{i\pi (\omega +\omega^{\prime })}{2(n_{c}+1)}}$ is multiplied both the numerator and denominator of the first exponential term, and $e^{-\frac{i\pi (\omega -\omega^{\prime })}{2(n_{c}+1)}}$ is multiplied on the top and bottom of the second term, producing

(A46) S=(−1)ω′+1Re−eiπ(ω+ω′)2(nc+1)−e−iπ(ω+ω′)2(nc+1)eiπ(ω+ω′)2(nc+1)+e−iπ(ω+ω′)2(nc+1)+eiπ(ω−ω′)2(nc+1)−e−iπ(ω−ω′)2(nc+1)eiπ(ω−ω′)2(nc+1)+e−iπ(ω−ω′)2(nc+1).

Now, both terms in brackets have a sine in the numerator and a cosine in the denominator. Since $e^{ix}-e^{-ix}=2i\sin x$ and $e^{ix}+e^{-ix}=2\cos x$, S is

(A47) S=(−1)ω′+1Re−2isin(ω+ω′)π2(nc+1)2cos(ω+ω′)π2(nc+1)+2isin(ω−ω′)π2(nc+1)2cosω−ω′)π2(nc+1).

Because each term inside the Re operation is purely imaginary this is identically 0. Therefore, the matrix element $t_{\omega \omega^{\prime }}$ matrix *Y* will be zero everywhere except those elements where $\omega +\omega^{\prime }=n_{c}+1$. These elements in the matrix could not be determined through this method because they cause a singularity in a denominator.

To show the behavior of the elements of $t_{\omega \omega^{\prime }}$ when $\omega =\omega^{\prime }$, one must backtrack to the general form of the sum S, which now is

(A48) $$S=2\delta_{\omega ,n_{c}+1-\omega^{\prime }}\sum_{k=1}^{n_{c}}{\left(-1\right)}^{k+1}\sin\left(\frac{\pi \omega k}{n_{c}+1}\right)\sin\left(\frac{\pi \omega^{\prime }\left(n_{c}+1-k\right)}{n_{c}+1}\right),$$

and substitute $\omega =n_{c}+1-\omega^{\prime }$ to obtain

(A49) $$S=2\delta_{\omega ,n_{c}+1-\omega^{\prime }}\sum_{k=1}^{n_{c}}{\left(-1\right)}^{k+1}\sin\left(\frac{\pi (n_{c}+1-\omega^{\prime })k}{n_{c}+1}\right)\sin\left(\frac{\pi \omega^{\prime }\left(n_{c}+1-k\right)}{n_{c}+1}\right).$$

It is useful to break the arguments of the sines into separate terms to look for identities. This gives

(A50) $$S=\delta_{\omega ,n_{c}+1-\omega^{\prime }}\sum_{k=1}^{n_{c}}{\left(-1\right)}^{k+1}\sin\left(\pi k-\frac{\pi \omega^{\prime }k}{n_{c}+1}\right)\sin\left(\pi \omega^{\prime }-\frac{\pi \omega^{\prime }k}{n_{c}+1}\right).$$

The trigonometric identity sin(u−v)=sinucosu−cosusinv allows this to be written as

(A51) $$\begin{array}{ccc} S & = & \delta_{\omega ,n_{c}+1-\omega^{\prime }}\sum_{k=1}^{n_{c}}{\left(-1\right)}^{k+1}\left[\sin\left(\pi k\right)\cos\left(\frac{\pi \omega^{\prime }k}{n_{c}+1}\right)-\cos\left(\pi k\right)\sin\left(\frac{\pi \omega^{\prime }k}{n_{c}+1}\right)\right]\\ & & \times \left[\sin\left(\pi \omega^{\prime }\right)\cos\left(\frac{\pi \omega^{\prime }k}{n_{c}+1}\right)-\cos\left(\pi \omega^{\prime }\right)\sin\left(\frac{\pi \omega^{\prime }k}{n_{c}+1}\right)\right]. \end{array}$$

Because *k* and $\omega^{\prime }$ are both integers, this reduces to

(A52) $$\begin{array}{lll} S & = & \delta_{\omega ,n_{c}+1-\omega^{\prime }}\sum_{k=1}^{n_{c}}{\left(-1\right)}^{k+1}\left[{\left(-1\right)}^{k+1}\sin\left(\frac{\pi \omega^{\prime }k}{n_{c}+1}\right)\right]\left[{\left(-1\right)}^{\omega^{\prime }+1}\sin\left(\frac{\pi \omega^{\prime }k}{n_{c}+1}\right)\right] \end{array}$$

(A53) $$\begin{array}{ccc} & = & \delta_{\omega ,n_{c}+1-\omega^{\prime }}{\left(-1\right)}^{\omega^{\prime }-1}\sum_{k=1}^{n_{c}}\sin^{2}\left(\frac{\pi \omega^{\prime }k}{n_{c}+1}\right), \end{array}$$

where the change in signs occurs via ${\left(-1\right)}^{k+1}{\left(-1\right)}^{k+1}{\left(-1\right)}^{\omega^{\prime }+1}={\left(-1\right)}^{2k}{\left(-1\right)}^{3}{\left(-1\right)}^{\omega^{\prime }}={\left(-1\right)}^{\omega^{\prime }+1}={\left(-1\right)}^{\omega^{\prime }-1}$. Now, the trig identity $2\sin^{2}\left(u\right)=1-\cos\left(2u\right)$ is used to give

(A54) $$S=\delta_{\omega ,n_{c}+1-\omega^{\prime }}{\left(-1\right)}^{\omega^{\prime }-1}\sum_{k=1}^{n_{c}}\left[1-\cos\left(\frac{2\pi \omega^{\prime }k}{n+1}\right)\right],$$

which has been seen before in solving the normalization of $u_{ql}$, where the cosine sum is −1, and so we have

(A55) $$S={\left(-1\right)}^{\omega^{\prime }-1}\left(n_{c}+1\right)\delta_{\omega ,n_{c}+1-\omega^{\prime }}.$$

The matrix elements can thus be written as

(A56) $$t_{\omega ,\omega^{\prime }}=\frac{i^{n_{c}+3}}{n_{c}+1}S=\frac{i^{n_{c}+3}}{n_{c}+1}{\left(-1\right)}^{\omega^{\prime }-1}\left(n_{c}+1\right)\delta_{\omega ,n_{c}+1-\omega^{\prime }}.$$

Recalling that $i^{n}=i^{n\;\mathrm{mod}\;4}$ allows this to be written as

(A57) $$t_{\omega ,\omega^{\prime }}=i^{n_{c}-1}{\left(-1\right)}^{\omega^{\prime }-1}\delta_{\omega ,n_{c}+1-\omega^{\prime }}.$$

This can also be written as

(A58) $$t_{\omega ,\omega^{\prime }}=i^{n_{c}-1}{\left(-1\right)}^{n_{c}-\omega }\delta_{\omega^{\prime },n_{c}+1-\omega }.$$

## Appendix E. Evaluating the Determinant and the Partition Function

The determinant of a cruciform matrix is easily determined by expanding by the first row of the matrix and then in each minor expanding by the last row. Then, each new minor is expanded by the first row and each of the resulting minors by their bottom rows. This repetition creates $\left\lfloor \frac{1}{2}\left(n+1\right)\right\rfloor$ factors of the type $\left(d_{11}d_{nn}-d_{1n}d_{n1}\right)$. The brackets ⌊x⌋ indicate the floor function, which gives the highest integer lower than *x*.

It is not immediately obvious that this is the correct expansion. This becomes clearer if we consider the general cruciform matrix $\bar{C}$

(A59) $$\left|\bar{C}\right|=\left(\begin{array}{cccccc} d_{11} & & & & & d_{1,n}\\ & d_{22} & & & d_{2,n-1} & \\ & & \ddots & ⋰ & & \\ & & ⋰ & \ddots & & \\ & d_{n-1,2} & & & d_{n-1,n-1} & \\ d_{n1} & & & & & d_{nn} \end{array}\right).$$

The expansion begins with the first row as

(A60) $$\begin{array}{cc} \left|\bar{C}\right|=d_{11} & \left|\begin{array}{ccccc} d_{22} & & & d_{2,n-1} & 0\\ & \ddots & ⋰ & & \\ & ⋰ & \ddots & & \\ d_{n-1,2} & & & d_{n-1,n-1} & \\ 0 & & & & d_{nn} \end{array}\right|\\ & +{\left(-1\right)}^{n-1}d_{1,n}\left|\begin{array}{ccccc} 0 & d_{22} & & & d_{2,n-1}\\ & & \ddots & ⋰ & \\ & & ⋰ & \ddots & \\ & d_{n-1,2} & & & d_{n-1,n-1}\\ d_{n1} & & & & 0 \end{array}\right|, \end{array}$$

where the only two elements in row one are multiplied by their minors. A factor of ${\left(-1\right)}^{n-1}$ is necessary to obtain the correct sign since it depends on the number of rows in the matrix. Then the remaining minors are expanded by their bottom rows. Note that this minor has n−1 rows, since its first row was eliminated,

(A61) $$\begin{array}{cc} \left|\bar{C}\right|=d_{11}d_{nn} & \left|\begin{array}{cccc} d_{22} & & & d_{2,n-1}\\ & \ddots & ⋰ & \\ & ⋰ & \ddots & \\ d_{n-1,2} & & & d_{n-1,n-1} \end{array}\right|\\ & +{\left(-1\right)}^{n-2}{\left(-1\right)}^{n-1}d_{1n}d_{n1}\left|\begin{array}{cccc} d_{22} & & & d_{2,n-1}\\ & \ddots & ⋰ & \\ & ⋰ & \ddots & \\ d_{n-1,2} & & & d_{n-1,n-1} \end{array}\right|. \end{array}$$

Notice that the second term in this expansion $d_{n1}$ was in the n−1 row and first column and so there is an additional factor of ${\left(-1\right)}^{n-2}$. In general, when taking a determinant, a negative sign is included with any element whose row and column positions sum to an odd number. The first term in the expansion of $|\bar{C}|$ will always be positive because the subscripts of the elements on the main diagonal always sum to an even number. The sums of the row and column numbers of the elements on the antidiagonal will depend on whether *n* is odd or even. However, if the matrix $\bar{C}$ has an odd number of rows and columns, then the minor obtained by expanding by a row will have an even number of rows and columns and vice versa. Therefore, either the element in the position $d_{1n}$ will have a negative sign or the one in position $d_{n1}$ will have a negative sign, but they will not both have one. The sign of the second term is then always negative, since ${\left(-1\right)}^{n-1}{\left(-1\right)}^{n-2}=-1$. After factoring out the remaining determinant, the expansion by the first row can be written as

(A62) $$\left|\bar{C}\right|=\left(d_{11}d_{nn}-d_{1n}d_{n1}\right)\left|\begin{array}{cccc} d_{22} & & & d_{2,n-1}\\ & \ddots & ⋰ & \\ & ⋰ & \ddots & \\ d_{n-1,2} & & & d_{n-1,n-1} \end{array}\right|.$$

The remaining determinant is expanded in the same way. This process is repeated until the matrix is fully expanded. The only thing to be aware of at this point is that if the matrix has odd dimension, the final remaining determinant will be of a single term.

The square of the partition function involves the evaluation of a cruciform matrix as

(A63) $$Z^{2}=\det\left[B\right]=\prod_{W=1}^{n_{r}}\det\Lambda_{W},$$

where the transformed matrix $\Lambda_{W}=X+2i\sin\left(\theta_{W}\right)Y$ is

(A64) $$\Lambda_{W}=\left(\begin{array}{cccccc} 2ix\sin(\varphi_{1}) & & & & & {\left(-1\right)}^{n_{c}-1}\xi )\\ & 2ix\sin(\varphi_{2}) & & & {\left(-1\right)}^{n_{c}-2}\xi & \\ & & \ddots & ⋰ & & \\ & & ⋰ & \ddots & & \\ & {\left(-1\right)}^{1}\xi & & & 2ix\sin(\varphi_{n_{c}-1}) & \\ {\left(-1\right)}^{0}\xi & & & & & 2ix\sin(\varphi_{n_{c}}) \end{array}\right),$$

where $\xi =2i^{n_{c}}y\sin\theta_{W}$. The first iteration in the expansion of this cruciform matrix $\Lambda_{W}$ is

(A65) $$\begin{array}{cc} \det\Lambda_{W}=\{[2ix & \sin\left(\varphi_{1}\right)]\left[2ix\sin\left(\varphi_{n_{c}}\right)\right]-\left[{\left(-1\right)}^{n_{c}-1}\xi \right]\left[{\left(-1\right)}^{0}\xi \right]\}\times \\ & \times \left|\begin{array}{cccc} 2ix\sin(\varphi_{2}) & & & {\left(-1\right)}^{n_{c}-2}\xi \\ & \ddots & ⋰ & \\ & ⋰ & \ddots & \\ {\left(-1\right)}^{1}\xi & & & 2ix\sin(\varphi_{n_{c}-1}) \end{array}\right|. \end{array}$$

A second iteration gives

(A66) $$\begin{array}{cc} \det\Lambda_{W}=\{[2ix & \sin\left(\varphi_{1}\right)]\left[2ix\sin\left(\varphi_{n_{c}}\right)\right]-\left[{\left(-1\right)}^{n_{c}-1}\xi \right]\left[{\left(-1\right)}^{0}\xi \right]\}\\ & \times \{\left[2ix\sin\left(\varphi_{2}\right)\right]\left[2ix\sin\left(\varphi_{n_{c}-1}\right)\right]-\left[{\left(-1\right)}^{n_{c}-2}\xi \right]\left[{\left(-1\right)}^{1}\xi \right]\}\\ & \times \left|\begin{array}{cccc} 2ix\sin(\varphi_{3}) & & & {\left(-1\right)}^{n_{c}-3}\xi \\ & \ddots & ⋰ & \\ & ⋰ & \ddots & \\ {\left(-1\right)}^{2}\xi & & & 2ix\sin(\varphi_{n_{c}-2}) \end{array}\right|, \end{array}$$

which is enough to see the pattern in the terms in the resulting product. In general, this determinant of the eigenvalue matrix reduces to the form

(A67) $$\begin{array}{cc} \det\Lambda_{W}=\prod_{s=1}^{\left\lfloor \frac{n}{2}\right\rfloor } & \left\{\left[2ix\sin\left(\varphi_{s}\right)\right]\left[2ix\sin\left(\varphi_{n_{c}+1-s}\right)\right]-{\left(-1\right)}^{n_{c}-s}{\left(-1\right)}^{s-1}\xi^{2}\right\}\\ & \times \left\{\begin{array}{cc} 1, & n\;\mathrm{even}\\ 2ix\sin\left(\varphi_{\frac{\left(n_{c}+1\right)}{2}}\right)+{\left(-1\right)}^{n_{c}-\frac{\left(n_{c}+1\right)}{2}}\xi , & n_{c}\;\mathrm{odd} \end{array}\right. \end{array},$$

where *s* is a placeholder positive integer for now. When $n_{c}$ is odd, the center term of the matrix is multiplied after the product is taken. This is what becomes last term in the odd expansion.

Ideally what is needed is a product of *ω* over the entire matrix $n_{c}$, but the extra term in the middle for odd matrices disallows a single product expression over *ω*. Recall that *ω* was the original subscript to φ and that *s* is just a placeholder. To make the change to a product over *ω*, the expression must be rewritten. We substitute ξ and pull the factor of $i^{\left(n_{c}-1\right)}$ out of the square in the second term. Then, a factor of $i^{2}$ is pulled out of all of the terms. This leaves

(A68) $$\begin{array}{ccc} |\Lambda_{W}| & = & \prod_{s=1}^{\left\lfloor \frac{n_{c}}{2}\right\rfloor }i^{2}\left\{\left[2x\sin\left(\varphi_{s}\right)\right]\left[2x\sin\left(\varphi_{n_{c}+1-s}\right)\right){]-\left(-1\right)}^{n_{c}-1}{\left(i\right)}^{2(n_{c}-2)}{\left[2iy\sin\left(\theta_{W}\right)\right]}^{2}\right\}\\ & & \times \left\{\begin{array}{cc} 1, & n_{c}\;\mathrm{even}\\ \left[2ix\sin\left(\varphi_{\frac{\left(n_{c}+1\right)}{2}}\right)+{\left(-1\right)}^{\frac{\left(n_{c}-1\right)}{2}}2i^{n_{c}}y\sin\left(\theta_{W}\right)\right], & n_{c}\;\mathrm{odd} \end{array}\right.. \end{array}$$

Because of the behavior of *i* when exponentiated, the sign terms inside the bracket reduce via ${\left(-1\right)}^{n_{c}-1}{\left(i\right)}^{2(n_{c}-2)}={\left(-1\right)}^{n_{c}-1}{\left(-1\right)}^{n_{c}}=-1$. The sign will always be −1 since $i^{2}=-1$. Therefore, $|\Lambda_{W}|$ can be written as

(A69) $$\begin{array}{ccc} |\Lambda_{W}| & = & {\left(-1\right)}^{\left\lfloor \frac{n_{c}}{2}\right\rfloor }\prod_{s=1}^{\left\lfloor \frac{n_{c}}{2}\right\rfloor }\left\{\left[2x\sin\left(\varphi_{s}\right)\right]\left[2x\sin\left(\varphi_{n_{c}+1-s}\right)\right]+{\left[2iy\sin\left(\theta_{W}\right)\right]}^{2}\right\}\\ & & \times \left\{\begin{array}{cc} 1, & n_{c}\;\mathrm{even}\\ \left[2ix\sin\left(\varphi_{\frac{\left(n_{c}+1\right)}{2}}\right)+{\left(-1\right)}^{\frac{\left(n_{c}-1\right)}{2}}2i^{n_{c}}y\sin\left(\theta_{W}\right)\right], & n_{c}\;\mathrm{odd} \end{array}\right., \end{array}$$

where $i^{2}$ has been moved outside of the product.

The next step in working toward a sum over *ω* is to factor the first bracketed term. This yields

(A70) $$\begin{array}{cc} |\Lambda_{W}|={\left(-1\right)}^{\left\lfloor \frac{n_{c}}{2}\right\rfloor }\prod_{s=1}^{\left\lfloor \frac{n_{c}}{2}\right\rfloor } & \left\{\left[2x\sin\left(\varphi_{s}\right)+2iy\sin\left(\theta_{\omega }\right)\right]\left[2x\sin\left(\varphi_{n_{c}+1-s}\right)+2iy\sin\left(\theta_{W}\right)\right]\right\}\\ & \times \left\{\begin{array}{cc} 1, & n_{c}\;\mathrm{even}\\ \left[2ix\sin\left(\varphi_{\frac{\left(n_{c}+1\right)}{2}}\right)+{\left(-1\right)}^{\frac{\left(n_{c}-1\right)}{2}}2i^{n_{c}}y\sin\left(\theta_{W}\right)\right], & n_{c}\;\mathrm{odd} \end{array}\right. \end{array},$$

where *s* skips over $\frac{n_{c}+1}{2}$ in the odd case for the moment. To arrive at this factorization, it is necessary to realize, from Equation (A70), that $\sin\left(\varphi_{s}\right)=-\sin\left(\varphi_{n_{c}+1-s}\right)$, which makes the cross terms cancel. We will show this now. Since $\varphi_{s}=\frac{\pi }{2}+\frac{\pi s}{n_{c}+1}$,

(A71) $$\sin\left(\varphi_{n_{c}+1-s}\right)=\sin\left(\frac{\pi }{2}+\frac{\pi (n_{c}+1-s)}{n_{c}+1}\right).$$

After splitting up the fraction this is

(A72) $$\sin\left(\varphi_{n_{c}+1-s}\right)=\sin\left(\frac{\pi }{2}+\frac{\pi (n_{c}+1)}{n_{c}+1}-\frac{\pi s}{n_{c}+1}\right).$$

which becomes

(A73) $$\sin\left(\varphi_{n_{c}+1-s}\right)=\sin\left(\frac{\pi }{2}+\pi -\frac{\pi s}{n_{c}+1}\right).$$

Through the trig identity sin(u±v)=sinucosvs.±cosusinv, the expression becomes

(A74) $$\sin\left(\varphi_{n_{c}+1-s}\right)=\sin\left(\frac{\pi }{2}\right)\cos\left(\pi -\frac{\pi s}{n_{c}+1}\right)+\cos\left(\frac{\pi }{2}\right)\sin\left(\pi -\frac{\pi s}{n_{c}+1}\right).$$

This is quickly reduced to

(A75) $$\sin\left(\varphi_{n_{c}+1-s}\right)=\cos\left(\pi -\frac{\pi s}{n_{c}+1}\right).$$

Then by cos(u±v)=cosucosvs.∓sinusinv the relationship is expressed as

(A76) $$\sin\left(\varphi_{n_{c}+1-s}\right)=\cos\left(\pi \right)\cos\left(\frac{\pi s}{n_{c}+1}\right)+\sin\left(\pi \right)\sin\left(\frac{\pi s}{n_{c}+1}\right),$$

which reduces to

(A77) $$\sin\left(\varphi_{n_{c}+1-s}\right)=-\cos\left(\frac{\pi s}{n_{c}+1}\right).$$

A factor of $\sin\left(\frac{\pi }{2}\right)$ is introduced, and then the relationship sin(u±v)=sinucosvs.±cosusinv is used in reverse, giving

(A78) $$\sin\left(\varphi_{n_{c}+1-s}\right)=-\sin\left(\frac{\pi }{2}+\frac{\pi s}{n_{c}+1}\right)=\sin\left(\varphi_{s}\right).$$

This relationship causes all of the cross-terms to cancel out and allows the factoring of the first term in the product of $|\Lambda_{W}|$.

This relationship is also useful for reducing the extra term in the odd expansion. In the case where $s=\frac{n_{c}+1}{2}$, it is shown here that $\sin(\varphi_{s})=0$.

(A79) $$\sin\varphi_{s}=\sin\left(\varphi_{\frac{n_{c}+1}{2}}\right)=-\sin\left(\frac{\pi }{2}+\frac{\pi }{n_{c}+1}\frac{n_{c}+1}{2}\right)=\sin\left(\pi \right)=0.$$

The term $\left[2ix\sin\left(\varphi_{\frac{\left(n_{c}+1\right)}{2}}\right)+{\left(-1\right)}^{\frac{\left(n_{c}-1\right)}{2}}2i^{n_{c}}y\sin\left(\theta_{W}\right)\right]$ can now be easily manipulated. The first term is 0 due to the relationship above. Now the factor ${\left(-1\right)}^{\frac{\left(n_{c}-1\right)}{2}}i_{c}^{n}$ in front of the $\sin(\theta_{W})$ needs to be investigated. First we see that

(A80) $${\left(-1\right)}^{\frac{\left(n_{c}-1\right)}{2}}i^{n_{c}}={\left(i^{2}\right)}^{\frac{\left(n_{c}-1\right)}{2}}i^{n_{c}}={\left(i\right)}^{2n_{c}-n_{c}-1}i^{n_{c}}={\left(i\right)}^{2n_{c}}{\left(i\right)}^{-1}.$$

Then because $i^{-1}=-i$, and because this is the case where $n_{c}$ is odd, $i^{2n_{c}}=-1$, and the above product reduces to

(A81) $${\left(-1\right)}^{\frac{\left(n_{c}-1\right)}{2}}i^{n_{c}}=\left(-1\right)\left(-i\right)=i.$$

Now, the leftover term in the odd expansion can be written as $2iy\sin\left(\theta_{W}\right)$. The product can be written as a single product from ω=1 to $n_{c}$ instead of being split into cases and instead of going to $\left\lfloor \frac{n_{c}}{2}\right\rfloor$.

(A82) $$\begin{array}{c} |\Lambda_{W}{|=\left(-1\right)}^{\left\lfloor \frac{n_{c}}{2}\right\rfloor }2^{n_{c}}\prod_{\omega =1}^{n_{c}}\left[x\sin\left(\varphi_{\omega }\right)+iy\sin\left(\theta_{W}\right)\right] \end{array},$$

where a 2 has been factored out of each term in the product, giving a $2^{n_{c}}$ out front. The two different sines, $\sin(\varphi_{\omega })$ and $\sin(\varphi_{n_{c}+1-\omega })$, will take opposite signs as *ω* goes from 1 to $\left\lfloor \frac{n_{c}}{2}\right\rfloor$. In this form of $\Lambda_{W}$, the term $\sin(\varphi_{\omega })$ will take all of the values that were formerly split between the combination of the two sines because the product now goes to $n_{c}$.

The arguments in $\Lambda_{W}$ can be reduced slightly. By the trig identity sin(u±v)=sinucosvs.±cosusinv, any angle in the form $\left(\frac{\pi }{2}+\frac{\pi s}{n_{c}+1}\right)$ can be written as $\cos\left(\frac{\pi \omega }{n_{c}+1}\right)$. Combining this with the definition of the state function in Equation (A63) gives

(A83) $$Z^{2}=\prod_{W=1}^{n_{r}}{\left(-1\right)}^{\left\lfloor \frac{n_{c}}{2}\right\rfloor }2^{n_{c}}\prod_{\omega =1}^{n_{c}}\left[x\cos\left(\frac{\pi \omega }{n_{c}+1}\right)+iy\cos\left(\frac{\pi W}{n_{r}+1}\right)\right].$$

After pulling the terms between the products out, the state function is

(A84) $$Z^{2}={\left(-1\right)}^{n_{r}\left\lfloor \frac{n_{c}}{2}\right\rfloor }2^{n_{r}n_{c}}\prod_{W=1}^{n_{r}}\prod_{\omega =1}^{n_{c}}\left[x\cos\left(\frac{\pi \omega }{n+1}\right)+iy\cos\left(\frac{\pi W}{n_{r}+1}\right)\right].$$

Equation (A84) is the exact square of the partition function of a finite $n_{r}\times n_{c}$ lattice as a product of $n_{r}n_{c}$ terms. If both $n_{r}$ and $n_{c}$ are odd, then there is a term in the product for which $\omega =\frac{1}{2}\left(n_{c}+1\right)$ and $W=\frac{1}{2}\left(n_{r}+1\right)$, which means that both the *x* and *y* parts vanish identically. The whole product is then multiplied by zero, correctly reflecting the fact that a lattice with an odd number of points cannot be occupied solely by dimers. Therefore, the lattice is required to have an even number of points and either $n_{r}$ or $n_{c}$ or both must be even.

Because the entropy will eventually be required, it would be best to have a partition function that is not squared. If it is assumed that $n_{c}$ is even, the partition function can be manipulated into a more useable form. Holding $n_{c}$ even instead of $n_{r}$ is an arbitrary choice. Moving the 2 back inside the products makes the partition function take the form

(A85) $$Z^{2}={\left(-1\right)}^{n_{r}\left\lfloor \frac{n_{c}}{2}\right\rfloor }\prod_{W=1}^{n_{r}}\prod_{\omega =1}^{n_{c}}\left[2x\cos\left(\frac{\pi \omega }{n_{c}+1}\right)+2iy\cos\left(\frac{\pi W}{n_{r}+1}\right)\right].$$

In the case where $n_{r}$ is odd, and focusing only on the product over *W*, it is possible to write this as

(A86) $${Z_{\begin{array}{c} n_{c}=\mathrm{even}\\ n_{r}=\mathrm{odd} \end{array}}}^{2}={\left(-1\right)}^{n_{r}\left\lfloor \frac{n_{c}}{2}\right\rfloor }\prod_{W=1}^{n_{r}}\prod_{\omega =1}^{n_{c}}\left[a+2iy\cos\left(\frac{\pi W}{n_{r}+1}\right)\right],$$

where $a=2x\cos\left(\frac{\pi \omega }{n_{c}+1}\right)$. Comparing the factor with W=1 to the factor with $W=n_{r}-1$ in the cosine shows that one of those terms is the negative of the other via

(A87) $$\cos\left(\frac{\pi n_{r}}{n_{r}+1}\right)=\cos\left(\pi -\frac{\pi }{n_{r}+1}\right)=-\cos\left(\frac{\pi }{n_{r}+1}\right).$$

This relationship holds for the pair W=2 and $W=n_{r}-1$ and so on. Thus, for each term in the product, there will appear a complex conjugate of that term. The only exception is the unpaired term where $\cos\left(\frac{\pi n_{r}}{n_{r}+1}\right)=\cos\left(\frac{\pi }{2}\right)=0$, and so that term must be written separately. With the terms paired off, the product can be cut in half.

(A88) $${Z_{\begin{array}{c} n=\mathrm{even}\\ m=\mathrm{odd} \end{array}}}^{2}={\left(-1\right)}^{m\lfloor \frac{n}{2}\rfloor }\left\{\prod_{r=1}^{n}\prod_{q=1}^{\left\lfloor \frac{m}{2}\right\rfloor }\left[a^{2}-{\left(2iy\cos\left(\frac{\pi q}{m+1}\right)\right)}^{2}\right]\right\}\left\{\prod_{r=1}^{n}\left(a+0\right)\right\}.$$

Substituting *a* gives

(A89) $$\begin{array}{ccc} {Z_{\begin{array}{c} n_{c}=\mathrm{even}\\ n_{r}=\mathrm{odd} \end{array}}}^{2} & = & {\left(-1\right)}^{n_{r}\left\lfloor \frac{n_{c}}{2}\right\rfloor }\left\{\prod_{\omega =1}^{n_{c}}\prod_{W=1}^{\left\lfloor \frac{n_{r}}{2}\right\rfloor }\left[4x^{2}\cos^{2}\left(\frac{\pi \omega }{n_{c}+1}\right)+4y^{2}\cos^{2}\left(\frac{\pi W}{n_{r}+1}\right)\right]\right\}\\ & & \times \left\{\prod_{\omega =1}^{n_{c}}2x\cos\left(\frac{\pi \omega }{n_{c}+1}\right)\right\}. \end{array}$$

The last product reduces to $x^{n_{c}}{\left(-1\right)}^{\frac{n_{c}}{2}}$ via the identity $\prod_{k=1}^{n-1}\cos\frac{k\pi }{n}=\frac{\sin\frac{\pi n}{2}}{2^{n-1}}$, as can be seen here,

(A90) $$\prod_{\omega =1}^{n_{c}}2x\cos\left(\frac{\pi \omega }{n_{c}+1}\right)=2^{n_{c}}x^{n_{c}}\prod_{\omega =1}^{n_{c}}\cos\left(\frac{\pi \omega }{n_{c}+1}\right)=2^{n_{c}+1}x^{n_{c}}\frac{\sin\frac{\pi \left(n_{c}+1\right))}{2}}{2^{n_{c}}}=x^{n_{c}}{\left(-1\right)}^{\frac{n_{c}}{2}}.$$

The sign factor ${\left(-1\right)}^{\frac{n_{c}}{2}}$ may be combined with the already existing sign factor to cancel as ${\left(-1\right)}^{n_{r}\left\lfloor \frac{n_{c}}{2}\right\rfloor }{\left(-1\right)}^{\frac{n_{c}}{2}}=1$ when $n_{c}$ is even and $n_{r}$ is odd. Then,

(A91) $${Z_{\begin{array}{c} n_{c}=\mathrm{even}\\ n_{r}=\mathrm{odd} \end{array}}}^{2}=x^{n_{c}}\prod_{\omega =1}^{n_{c}}\prod_{W=1}^{\left\lfloor \frac{n_{r}}{2}\right\rfloor }\left[4x^{2}\cos^{2}\left(\frac{\pi \omega }{n_{c}+1}\right)+4y^{2}\cos^{2}\left(\frac{\pi W}{n_{r}+1}\right)\right].$$

Using the same sort of expansion over the $n_{c}$ as was used for the product over $n_{r}$ gives

(A92) $${Z_{\begin{array}{c} n_{c}=\mathrm{even}\\ n_{r}=\mathrm{odd} \end{array}}}^{2}=x^{n_{c}}\prod_{\omega =1}^{\frac{n_{c}}{2}}\prod_{W=1}^{\left\lfloor \frac{n_{r}}{2}\right\rfloor }{\left[4x^{2}\cos^{2}\left(\frac{\pi \omega }{n_{c}+1}\right)+4y^{2}\cos^{2}\left(\frac{\pi W}{n_{r}+1}\right)\right]}^{2}.$$

Equation (A84) is the exact partition function of a finite $n_{r}\times n_{c}$ lattice as a product of $n_{r}n_{c}$ terms. If both $n_{c}$ and $n_{r}$ are odd, then there is a term in the product for which $r\omega =\frac{1}{2}\left(n_{c}+1\right)$ and $W=\frac{1}{2}\left(n_{r}+1\right)$, which means that both the *x* and *y* parts vanish identically. The whole product is then multiplied by zero, correctly reflecting the fact that a lattice with an odd number of points can not be occupied solely by dimers. Therefore, the lattice is required to have an even number of points, and either $n_{r}$ or $n_{c}$ or both must be even.

In the case for which $n_{r}$ is even, the solution follows the same process. The key differences are that the product over $n_{r}$ will not have an unpaired term when $n_{r}$ is even and the sign term in *Z* reduces identically to 1. Thus, the partition function is

(A93) $$Z=2^{\frac{n_{c}}{2}\left\lfloor \frac{n_{r}}{2}\right\rfloor }\prod_{\omega =1}^{\frac{n_{c}}{2}}\prod_{W=1}^{\left\lfloor \frac{n_{r}}{2}\right\rfloor }\left[x^{2}\left(1+\cos\left(\frac{2\pi \omega }{n_{c}+1}\right)\right)+y^{2}\left(1+\cos\left(\frac{2\pi W}{n_{r}+1}\right)\right)\right]\times \left\{\begin{array}{cc} 1 & n_{r}\;\mathrm{even}\\ x^{\frac{n_{c}}{2}} & n_{r}\;\mathrm{odd} \end{array}\right..$$
